# Supplementary material for: Association between Reactogenicity and Immunogenicity in a Vaccinated Cohort with Two mRNA SARS-CoV-2 Vaccines at a High-Complexity Reference Hospital: A Post Hoc Analysis on Immunology Aspects of a Prospective Cohort Study
Source: Vaccines (Basel). 2024 Jun 17;12(6):665. doi: 10.3390/vaccines12060665 (PMC11209257; doi:10.3390/vaccines12060665)
Supplement: Supplementary file 1 [file vaccines-12-00665-s001.zip › vaccines-3008116-supplementary.pdf]

ELECTRONIC SUPPLEMENTARY MATERIAL

Journal: Vaccines

Association between reactogenicity and immunogenicity in a vaccinated cohort with two mRNA SARS-CoV-2 vaccines at a high-complexity reference hospital: a *post-hoc* analysis on immunology aspects of a prospective cohort study

Joaquín Sáez-Peñataro[1*], Gonzalo Calvo,[1] Juan Bascuas[1], Maria Mar Mosquera[2], Maria Ángeles Marcos[2,3], Natalia Egri[4], Ferran Torres[5]

Author´s affiliations:

[1] Medicines Division, Department of Clinical Pharmacology, Hospital Clínic de Barcelona, Institut d'Investigacions Biomèdiques August Pi i Sunyer (IDIBAPS), Universitat de Barcelona, Barcelona, Spain

[2] Microbiology Department, Hospital Clinic, Institute for Global Health, University of Barcelona, Barcelona, Spain

[3] CIBERINF, Madrid, Spain

[4] Immunology Department, Hospital Clínic de Barcelona, Institut d'Investigacions Biomèdiques August Pi i Sunyer (IDIBAPS), Universitat de Barcelona, Barcelona, Spain

[5] Department of Biostatistics, Autonomous University of Barcelona, Barcelona, Spain

Corresponding author:

Joaquín Sáez-Peñataro (Email contact: jsaez@clinic.cat)

Phone number: +34 93 227 5400 extension: 1451

CONTENTS

Tables:

i) Measurement of antibodies and T-cell response. Analytical methods
ii) Main analysis and sensitivity analyses:
ii.i Main analysis:
Table S1: Assessment of antibody positiveness (positive-negative titres)
Table S2: Assessment of antibody positiveness (positive-negative titres): solicited and unsolicited AR
Table S3: Assessment of antibody positiveness (positive-negative titres): severe solicited AR
Table S4: Assessment of antibody titres (antibody titres above-below median)
Table S5: Assessment of antibody titres (antibody titres above-below median): solicited and unsolicited AR
Table S6: Assessment of antibody titres (antibody titres above-below median): severe solicited AR
Figure S1: Summary of results of immunogenicity and reactogenicity of the main population

ii.ii Sensitivity analysis 1:
Table S7: Characteristics of subpopulation 1
Table S8: Assessment of antibody positiveness (positive-negative titres)
Table S9: Assessment of antibody positiveness (positive-negative titres): solicited and unsolicited AR
Table S10: Assessment of antibody positiveness (positive-negative titres): severe solicited AR
Table S11: Assessment of antibody titres (antibody titres above-below median)
Table S12: Assessment of antibody titres (antibody titres above-below median): solicited and unsolicited AR
Table S13: Assessment of antibody titres (antibody titres above-below median): severe solicited AR
Table S14: Cellular immunogenicity assessment
Table S15: Cellular immunogenicity assessment: solicited and unsolicited AR
Table S16: Cellular immunogenicity assessment: severe solicited AR
Figure S2: Summary of results of immunogenicity and reactogenicity of sensitivity analysis 1

ii.iii Sensitivity analysis 2:
Table S17: Characteristics of subpopulation 2
Table S18: Assessment of antibody positiveness (positive-negative titres)
Table S19: Assessment of antibody positiveness (positive-negative titres): solicited and unsolicited AR
Table S20: Assessment of antibody positiveness (positive-negative titres): severe solicited AR
Table S21: Assessment of antibody titres (antibody titres above-below median):
Table S22: Assessment of antibody titres (antibody titres above-below median): solicited and unsolicited AR
Table S23: Assessment of antibody titres (antibody titres above-below median): severe solicited AR
Figure S3: Summary of results of immunogenicity and reactogenicity of sensitivity analysis 2

ii.iv Exploratory analysis:
Table S24: Reactogenicity and immunogenicity by vaccine and population types
Figure S4: Comparison of vaccine types
Figure S5: Comparison of population types

iv Table S25: STROBE Checklist

Measurement of antibodies and T-cell response. Analytical methods
Humoral immunogenicity

Levels of neutralizing and anti-spike antibodies (IgG + IgM) were measured on the Cobas platform (Roche©), based on semiquantitative measurement of antibody titres through serological enhanced "electrochemiluminiscence", on a scale that ranges from 0 to >10. Results >1.0 were considered positive antibody titres. IgG titres were measured on samples that tested positive in the Cobas platform in a subset of patients, as per local clinical practice protocols (Akashi Y et al, 2022). During the vaccination campaign the measurement method changed, and IgG titres were measured in the Atellica Analyzer (Siemens Healthineers©) (Florin L et al, 2021), with a quantitative scale that ranged from 0.5 to >150 U/ml. IgG were measured only in a subset of the total vaccinated population (N=322 vaccinees).

Cellular immunogenicity

SARS-CoV-2-specific T-cell response was measured by interferon-gamma ELISPOT assay measured from isolated PBMCs, as described by Egri N et al (Egri N et al., 2022).

In summary, venous blood samples were obtained from the forearm utilizing a butterfly needle connected to heparinized and serum-containing tubes. Peripheral blood mononuclear cells (PBMCs) were extracted from the blood samples through Ficoll-Paque density gradient centrifugation. Stimulation experiments were performed using 2 × 105 PBMCs cultured in X-VIVO™15 medium supplemented with 10% heat-inactivated AB serum and PepTivator® SARS-CoV-2 Prot_S and N peptide pools at a concentration of mg/ml (Miltenyi Bio-tec®). The diluent utilized was PBS+DMSO with a final DMSO concentration of 1%. For the ELISpot negative control, X-VIVO 15 medium with 20% DMSO to a final concentration of 1% was employed. Negative control wells were devoid of peptides, while positive control wells contained mAb CD3-2 from the kit. Cells were then cultured overnight (16–20 h) at 37°C with 5% CO2 in precoated anti-IFN-g MSIP white plates (Human IFNg ELISpotPRO kit (ALP) (Mabtech) Ref: 3420-2AST-2, Mabtech®). Following incubation, plates were washed five times with PBS (Sigma-Aldrich) and incubated for 2 h at room temperature with horseradish peroxidase (HRP)-conjugated anti-IFN-g detection antibody (1 mg/ml; clone mAb-7B6-1; Mabtech®).

After five additional washes with PBS, tetra-methylbenzidine (TMB) substrate was added, and spots were enumerated using an automated ELISpot Reader System (Autoimmun Diagnostika GmbH®). Positive peptide-specific responses were quantified by subtracting spots in unstimulated wells from peptide-stimulated wells, with results expressed as SFU (Spot Forming Units)/2x105 PBMCs. SARS-CoV-2-specific spots were determined by spot increment, defined as stimulated spot numbers ≥6 SFU/2 × 105 PBMCs. This threshold was established by calculating the mean ± 2 standard deviations in a cohort of healthy donors obtained prior to the onset of the SARS-CoV-2 pandemic. Spot counting was conducted automatically and was manually verified in all instances.

References:

Akashi Y, Horie M, Kiyotaki J, et al. Clinical Performance of the cobas Liat SARS-CoV-2 & Influenza A/B Assay in Nasal Samples. Mol Diagn Ther. 2022;26(3):323-331. doi:10.1007/s40291-022-00580-8

Florin L, Maelegheer K, Vandewal W, Bernard D, Robbrecht J. Performance Evaluation of the Siemens SARS-CoV-2 Total Antibody and IgG Antibody Test. Lab Med. 2021;52(6):e147-e153. doi:10.1093/labmed/lmab027

Egri N, Olivé V, Hernández-Rodríguez J, & et al. (2022). CoVITEST: A Fast and Reliable Method to Monitor Anti-SARS-CoV-2 Specific T Cells From Whole Blood. Front Immunol, 13, 848586.

ii. Main analysis and sensitivity analyses

ii.i Main analysis

Table S1 Assessment of antibody positiveness (positive-negative titres)

Variable	Antibody positiveness											
	Negative antibody titres (n= 64)						Positive antibody titres (n= 151)					
	After 1st vaccine dose	p-value	After 2nd vaccine dose	p-value	After any dose	p-value	After 1st vaccine dose	p-value	After 2nd vaccine dose	p-value	After any dose	p-value
**Proportions of AR**												
Development of any AR (n, %)	43 (67.2)	0.891	37 (57.8)	0.408	52 (81.3)	0.368	100 (66.2)	0.891	78 (51.7)	0.408	130 (86.1)	0.368
Development of any mild AR (n, %)	19 (29.7)	0.071	8 (12.5)	0.905	26 (40.6)	0.100	28 (18.5)	0.071	18 (11.9)	0.905	44 (29.1)	0.100
Development of any moderate AR (n, %)	20 (31.3)	0.586	26 (40.6)	0.250	38 (59.4)	0.494	53 (35.1)	0.586	49 (32.5)	0.250	82 (54.3)	0.494
Development of any severe AR (n, %)	10 (15.6)	0.296	16 (25.0)	0.774	22 (34.4)	0.775	33 (21.9)	0.296	35 (23.2)	0.774	55 (36.4)	0.775
**Maximum intensity**												
Grade 1 (n, %)	13 (31.0)	0.339	4 (11.4)	0.890	6 (11.8)	0.497	21 (22.8)	0.339	11 (14.5)	0.890	22 (17.9)	0.497
Grade 2 (n, %)	19 (45.2)		15 (42.9)		23 (45.1)		38 (41.3)		30 (39.5)		46 (37.4)	
Grade 3 (n, %)	10 (23.8)		16 (45.7)		22 (43.1)		33 (35.9)		35 (46.1)		55 (44.7)	
Likert score Mean (SD)	4.79 (2.04)	0.106	6.09 (2.07)	0.858	5.92 (2.12)	0.872	5.44 (2.19)	0.106	6.22 (2.02)	0.858	5.92 (2.21)	0.872

Antibody titres according to proportions (rate) of AR and intensity of AR. AR: adverse reaction; Ab: antibody. Results are provided as absolute and relative (%) numbers for each variable. Statistically significant results are marked in bold type

Table S2 Assessment of antibody positiveness (positive-negative titres): solicited and unsolicited AR

**Adverse reactions (PT MedDRA term)**	**Humoral immunogenicity**				**[a]p-value**	
	**Negative antibody titres**		**Positive antibody titres**			
	**After any dose**		**After any dose**			
	**After 1st dose**	**After 2nd dose**	**After 1st dose**	**After 2nd dose**		
**Solicited adverse reactions**						
SOC: *General disorders and administration site conditions*						
Injection site pain	31 (48.4)		56 (37.1)		0.121	
PT: Injection site pain	23 (35.9)	11 (17.2)	47 (31.1)	21 (13.9)	0.491	0.537
Fatigue/astenia	16 (25.0)		27 (17.9)		0.233	
PT: Fatigue	4 (6.3)	12 (18.8)	8 (5.3)	21 (13.9)	0.781	0.368
Fever	17 (26.6)		46 (30.5)		0.566	
PT: Pyrexia	4 (6.3)	13 (20.3)	20 (13.2)	35 (23.2)	0.136	0.645
Malaise	4 (6.3)		24 (15.9)		0.055	
PT: Malaise	1 (1.6)	3 (4.7)	4 (2.6)	19 (12.6)	0.629	0.081
Chills	3 (4.7)		11 (7.3)		0.480	
PT: Chills	0 (0)	3 (4.7)	4 (2.6)	7 (4.6)	0.189	0.987
Injection site redness	3 (4.7)		4 (2.6)		0.441	
PT: Application site redness	2 (3.1)	2 (3.1)	2 (1.3)	2 (1.3)	0.372	0.372

Adverse reactions (PT MedDRA term)	Humoral immunogenicity				[a]p-value	
	Negative antibody titres		Positive antibody titres			
	After any dose		After any dose			
	After 1st dose	After 2nd dose	After 1st dose	After 2nd dose		
SOC: *Musculoskeletal and connective tissue disorders*						
Arm pain *PT: Pain in extremity*	15 (23.4)		38 (25.2)		0.788	
	9 (14.1)	8 (12.5)	27 (17.9)	14 (9.3)	0.493	0.475
Muscle pain *PT: Myalgia*	12 (18.8)		29 (19.2)		0.938	
	2 (3.1)	10 (15.6)	7 (4.6)	24 (15.9)	0.613	0.961
Joint pain *PT: Arthralgia*	5 (7.8)		13 (8.6)		0.847	
	0 (0)	5 (7.8)	1 (0.7)	12 (7.9)	0.514	0.973
Shoulder pain *PT: Musculoskeletal pain*	3 (4.7)		6 (4.0)		0.811	
	2 (3.1)	1 (1.6)	4 (2.6)	1 (0.7)	0.770	0.530
SOC: *Injury, poisoning and procedural complications*						
Injection site swelling *PT: Application site swelling*	5 (7.8)		10 (6.6)		0.754	
	3 (4.7)	2 (3.1)	9 (6.0)	2 (1.3)	0.710	0.372
SOC: Vaccination site pruritus						
Injection site pruritus *PT: Vaccination site pruritus*	1 (1.6)		2 (1.3)		0.892	
	0 (0)	1 (1.6)	0 (0)	2 (1.3)	NA	0.892

Adverse reactions (PT MedDRA term)	Humoral immunogenicity				[a]p-value	
	Negative antibody titres		Positive antibody titres			
	After any dose		After any dose			
	After 1st dose	After 2nd dose	After 1st dose	After 2nd dose		
SOC: Nervous system disorders						
Headache *PT: Headache*	19 (29.7)		40 (26.5)		0.631	
	8 (12.5)	13 (20.3)	19 (12.6)	23 (15.2)	0.987	0.362
Facial paralysis *PT: Facial paralysis*	0 (0)		0 (0)		NA	
	0 (0)	0 (0)	0 (0)	0 (0)	NA	NA
Insomnia *PT: Insomnia*	2 (3.1)		2 (1.3)		0.372	
	0 (0)	2 (3.1)	0 (0)	2 (1.3)	NA	0.372
SOC: Gastrointestinal disorders						
Nausea *PT: Nausea*	2 (3.1)		5 (3.3)		0.944	
	1 (1.6)	1 (1.6)	4 (2.6)	1 (0.7)	0.629	0.530
Diarrhea *PT: Diarrhoea*	1 (1.6)		2 (1.3)		0.892	
	1 (1.6)	0 (0)	1 (0.7)	1 (0.7)	0.530	0.514
Vomiting *PT: Vomiting*	0 (0)		0 (0)		NA	
	0 (0)	0 (0)	0 (0)	0 (0)	NA	NA

**Adverse reactions (PT MedDRA term)**	**Humoral immunogenicity**				**[a]p-value**	
	**Negative antibody titres**		**Positive antibody titres**			
	**After any dose**		**After any dose**			
	**After 1st dose**	**After 2nd dose**	**After 1st dose**	**After 2nd dose**		
**Unsolicited adverse reactions**						
SOC: *Musculoskeletal and connective tissue disorders*						
Other musculoskeletal disorders	3 (4.7)		4 (2.6)		0.441	
PT: Musculoskeletal disorder	1 (1.6)	2 (3.1)	0 (0)	4 (2.6)	0.124	0.846
SOC: *Skin and subcutaneous tissue disorders*						
Petechia, ecchymosis	0 (0)		0 (0)		NA	
PT: *Ecchymosis*	0 (0)	0 (0)	0 (0)	0 (0)	NA	NA
SOC: *Nervous system disorders*						
Cognitive alteration	0 (0)		0 (0)		NA	
PT: Cognitive disorder	0 (0)	0 (0)	0 (0)	0 (0)	NA	NA
Alterations of smell and taste	0 (0)		1 (0.7)		0.514	
PT: Parosmia	0 (0)	0 (0)	0 (0)	1 (0.7)	NA	0.514
Paresthesia and hyperesthesia	1 (1.6)		2 (1.3)		0.892	
PT: Dysaesthesia	0 (0)	1 (1.6)	2 (1.3)	0 (0)	0.355	0.124
Presyncope, syncope and vasovagal syncope	0 (0)		0 (0)		NA	
PT: Syncope	0 (0)	0 (0)	0 (0)	0 (0)	NA	NA
Sleepiness, hypersomnia	0 (0)		0 (0)		NA	
PT: Hypersomnia	0 (0)	0 (0)	0 (0)	0 (0)	NA	NA

Adverse reactions (PT MedDRA term)	Humoral immunogenicity				[a]p-value	
	Negative antibody titres		Positive antibody titres			
	After any dose		After any dose			
	After 1st dose	After 2nd dose	After 1st dose	After 2nd dose		
Instability sensation, vertigo, sickness<br>*PT:*<br>*Dizziness*	1 (1.6)		2 (1.3)		0.892	
	0 (0)	1 (1.6)	1 (0.7)	1 (0.7)	0.514	0.530
Tremor<br>*PT:*<br>*Tremor*	0 (0)		0 (0)		NA	
	0 (0)	0 (0)	0 (0)	0 (0)	NA	NA
SOC: *Gastrointestinal disorders*						
Gastrointestinal disorders<br>*PT:*<br>*Gastrointestinal disorder*	0 (0)		1 (0.7)		0.514	
	0 (0)	0 (0)	1 (0.7)	0 (0)	0.514	NA
SOC: *Respiratory, thoracic and mediastinal disorders*						
Asthma<br>*PT:*<br>*Asthma*	0 (0)		0 (0)		NA	
	0 (0)	0 (0)	0 (0)	0 (0)	NA	NA
Rhinitis, nasal discharge<br>*PT:*<br>*Rhinitis*	0 (0)		1 (0.7)		0.514	
	0 (0)	0 (0)	0 (0)	1 (0.7)	NA	0.514

Adverse reactions (PT MedDRA term)	Humoral immunogenicity				[a]p-value	
	Negative antibody titres		Positive antibody titres			
	After any dose		After any dose			
	After 1st dose	After 2nd dose	After 1st dose	After 2nd dose		
SOC: *Investigations*						
Hypertension, hypotension<br>*PT:*<br>*Blood pressure abnormal*	**2 (3.1)**		**0 (0)**		**0.029**	
	0 (0)	**2 (3.1)**	0 (0)	**0 (0)**	NA	**0.029**
SOC: *General disorders and administration site conditions*						
Influenza-like symptoms<br>PT:<br>*Influenza like illness*	0 (0)		0 (0)		NA	
	0 (0)	0 (0)	0 (0)	0 (0)	NA	NA
Sensation of heat, sensation of cold<br>PT: *Temperature regulation disorder*	0 (0)		2 (1.3)		0.355	
	0 (0)	0 (0)	1 (0.7)	1 (0.7)	0.514	0.514
Chest pain<br>PT:<br>*Chest pain*	0 (0)		0 (0)		NA	
	0 (0)	0 (0)	0 (0)	0 (0)	NA	NA
Hiporexia, anorexia<br>PT:<br>*Decreased appetite*	0 (0)		0 (0)		NA	
	0 (0)	0 (0)	0 (0)	0 (0)	NA	NA
Inflammation in extremities other than the vaccinated arm<br>PT:<br>*Inflammation*	0 (0)		0 (0)		NA	
	0 (0)	0 (0)	0 (0)	0 (0)	NA	NA

Adverse reactions (PT MedDRA term)	Humoral immunogenicity				[a]p-value	
	Negative antibody titres		Positive antibody titres			
	After any dose		After any dose			
	After 1st dose	After 2nd dose	After 1st dose	After 2nd dose		
SOC: *Ear and labyrinth disorders*						
PT: *Ear pain*	0 (0)		0 (0)		NA	
	0 (0)	0 (0)	0 (0)	0 (0)	NA	NA
SOC: *Skin and subcutaneous tissue disorders*						
General pruritus PT: *Pruritus*	**2 (3.1)**		**0 (0)**		**0.029**	
	1 (1.6)	1 (1.6)	0 (0)	0 (0)	0.124	0.124
Sweat PT: *Cold sweat*	0 (0)		0 (0)		NA	
	0 (0)	0 (0)	0 (0)	0 (0)	NA	NA
SOC: *Immune system disorders*						
Immediate hypersensitivity, delayed hypersensitivity, exanthema, urticaria, rash PT: *Hypersensitivity*	1 (1.6)		1 (0.7)		0.530	
	0 (0)	1 (1.6)	1 (0.7)	0 (0)	0.514	0.124
SOC: *Cardiac disorders*						
Tachycardia PT: *Tachycardia*	0 (0)		1 (0.7)		0.514	
	0 (0)	0 (0)	0 (0)	1 (0.7)	NA	0.514

Adverse reactions (PT MedDRA term)	Humoral immunogenicity				[a]p-value	
	Negative antibody titres		Positive antibody titres			
	After any dose		After any dose			
	After 1st dose	After 2nd dose	After 1st dose	After 2nd dose		
SOC: *Blood and lymphatic system disorders*						
Lymphadenopathy<br>PT:<br>*Lymphadenopathy*	0 (0)		6 (4.0)		0.106	
	0 (0)	0 (0)	1 (0.7)	5 (3.3)	0.514	0.141
SOC: *Infections and infestations*						
Herpetic infection<br>PT:<br>*Herpes virus infection*	0 (0)		0 (0)		NA	
	0 (0)	0 (0)	0 (0)	0 (0)	NA	NA

Distribution of proportions of solicited and unsolicited AR are displayed by elicited qualitative humoral immunogenicity and dose; [a]chi-square test; AR: adverse reaction; SOC: System Organ Class; PT: Preferred term; NA: not applicable, p-value not calculable because of absence of valid values in both comparison groups; in bold type: statistically significant results. Results are provided as absolute and relative (%) numbers for each variable.

Table S3 Assessment of antibody positiveness (positive-negative titres): severe solicited AR

Adverse reactions (PT MedDRA term)	Humoral immunogenicity				[a]p-value	
	Negative antibody titres		Positive antibody titres			
	After any dose		After any dose			
	After 1st dose	After 2nd dose	After 1st dose	After 2nd dose		
**Solicited adverse reactions**						
SOC: *General disorders and administration site conditions*						
Injection site pain<br>*PT: Injection site pain*	3 (4.7)		15 (9.9)		0.204	
	3 (4.7)	0 (0)	13 (8.6)	3 (2.0)	0.316	0,256
Fatigue/asthenia<br>*PT:*<br>*Fatigue*	9 (14.1)		10 (6.6)		0.079	
	**3 (4.7)**	6 (9.4)	**1 (0.7)**	9 (6.0)	**0.046**	0.369
Fever<br>*PT:*<br>*Pyrexia*	0 (0)		7 (4.6)		0.080	
	0 (0)	0 (0)	3 (2.0)	5 (3.3)	0.136	0.141
Malaise<br>*PT:*<br>*Malaise*	**1 (1.6)**		**15 (9.9)**		**0.033**	
	0 (0)	1 (1.6)	1 (0.7)	13 (8.6)	0.514	0.056
Chills<br>*PT:*<br>*Chills*	0 (0)		0 (0)		NA	
	0 (0)	0 (0)	0 (0)	0 (0)	NA	NA
Injection site redness<br>*PT:*<br>*Application site redness*	0 (0)		1 (0.7)		0.514	
	0 (0)	0 (0)	1 (0.7)	0 (0)	0.514	NA

Adverse reactions (PT MedDRA term)	Humoral immunogenicity				[a]p-value	
	Negative antibody titres		Positive antibody titres			
	After any dose		After any dose			
	After 1st dose	After 2nd dose	After 1st dose	After 2nd dose		
SOC: *Musculoskeletal and connective tissue disorders*						
Arm pain *PT: Pain in extremity*	8 (12.5)		15 (9.9)		0.578	
	6 (9.4)	2 (3.1)	10 (6.6)	5 (3.3)	0.482	0.944
Muscle pain *PT: Myalgia*	12 (18.8)		29 (19.2)		0.938	
	2 (3.1)	10 (15.6)	7 (4.6)	24 (15.9)	0.613	0.961
Joint pain *PT: Arthralgia*	3 (4.7)		10 (6.6)		0.586	
	0 (0)	3 (4.7)	0 (0)	10 (6.6)	NA	0.586
Shoulder pain *PT: Musculoskeletal pain*	0 (0)		2 (1.3)		0.355	
	0 (0)	0 (0)	1 (0.7)	0 (0)	0.514	NA
SOC: *Injury, poisoning and procedural complications*						
Injection site swelling *PT: Application site swelling*	0 (0)		4 (2.6)		0.189	
	0 (0)	0 (0)	4 (2.6)	0 (0)	0.189	NA
SOC: Vaccination site pruritus						
Injection site pruritus *PT: Vaccination site pruritus*	0 (0)		0 (0)		NA	
	0 (0)	0 (0)	0 (0)	0 (0)	NA	NA

Adverse reactions (PT MedDRA term)	Humoral immunogenicity				[a]p-value	
	Negative antibody titres		Positive antibody titres			
	After any dose		After any dose			
	After 1st dose	After 2nd dose	After 1st dose	After 2nd dose		
SOC: Nervous system disorders						
Headache	6 (9.4)		17 (11.3)		0.683	
PT: Headache	2 (3.1)	5 (7.8)	6 (4.0)	11 (7.3)	0.764	0.893

Distribution of proportions of severe solicited and unsolicited AR are displayed by elicited qualitative humoral immunogenicity and dose; [a]chi-square test; AR: adverse reaction; SOC: System Organ Class; PT: Preferred term; NA: not applicable, p-value not calculable because of absence of valid values in both comparison groups; in bold type: statistically significant results. Results are provided as absolute and relative (%) numbers for each variable

Table S4 Assessment of antibody titres (antibody titres above-below median):

Variable	Antibody titres below median* (n= 93)						Antibody titres above median* (n= 122)					
	After 1st vaccine dose	p-value	After 2nd vaccine dose	p-value	After any dose	p-value	After 1st vaccine dose	p-value	After 2nd vaccine dose	p-value	After any dose	p-value
**Proportions of AR**												
Development of any AR (n, %)	58 (62.4)	0.261	51 (54.8)	0.729	75 (80.6)	0.155	85 (69.7)	0.261	64 (52.5)	0.729	107 (87.7)	0.155
Development of any mild AR (n, %)	22 (23.7)	0.578	14 (15.1)	0.245	33 (35.5)	0.424	25 (20.5)	0.578	12 (9.8)	0.245	37 (30.3)	0.424
Development of any moderate AR (n, %)	28 (30.1)	0.299	34 (36.6)	0.653	53 (57.0)	0.762	45 (36.9)	0.299	41 (33.6)	0.653	67 (54.9)	0.762
Development of any severe AR (n, %)	14 (15.1)	0.113	24 (25.8)	0.530	33 (35.5)	0.930	29 (23.8)	0.113	27 (22.1)	0.530	44 (36.1)	0.930
Maximum intensity												
Grade 1 (n, %)	15 (27.3)	0.386	6 (12.2)	0.840	9 (12.3)	0.501	19 (24.1)	0.386	9 (14.5)	0.840	19 (18.8)	0.501
Grade 2 (n, %)	26 (47.3)		19 (38.8)		31 (42.5)		31 (39.2)		26 (41.9)		38 (37.6)	
Grade 3 (n, %)	14 (25.5)		24 (49.0)		33 (45.2)		29 (36.7)		27 (43.5)		44 (43.6)	
Likert score Mean (SD)	5.09 (2.15)	0.489	6.19 (2.06)	0.896	6.07 (2.13)	0.414	5.33 (2.17)	0.489	6.17 (2.02)	0.896	5.80 (2.21)	0.414

Antibody titres (in median titres </> median) according to proportions (rate) of AR and intensity of AR. AR: adverse reaction; Ab: antibody. Results are provided as absolute and relative (%) numbers for each variable. Statistically significant results are marked in bold type, * Median value of antibody titres (P25, P75)= 7.69 (0.73,10.00) on a semiquantitative scale ranging from 0 to >10

Table S5 Assessment of antibody titres (antibody titres above-below median): solicited and unsolicited AR

Adverse reactions (PT MedDRA term)	Humoral immunogenicity				[a]p-value	
	Antibody titres below median* (n= 93)		Antibody titres above median* (n= 122)			
	After any dose		After any dose			
	After 1st dose	After 2nd dose	After 1st dose	After 2nd dose		
**Solicited adverse reactions**						
SOC: *General disorders and administration site conditions*						
Injection site pain	39 (41.9)		48 (39.3)		0.701	
PT: Injection site pain	29 (31.2)	16 (17.2)	41 (33.6)	16 (13.1)	0.707	0.404
Fatigue/asthenia	23 (24.7)		20 (16.4)		0.130	
PT: Fatigue	5 (5.4)	18 (19.4)	7 (5.7)	15 (12.3)	0.909	0.155
Fever	24 (25.8)		39 (32.0)		0.326	
PT: Pyrexia	**5 (5.4)**	19 (20.4)	**19 (15.6)**	29 (23.8)	**0.019**	0.560
Malaise	9 (9.7)		19 (15.6)		0.203	
PT: Malaise	1 (1.1)	8 (8.6)	4 (3.3)	14 (11.5)	0.288	0.491
Chills	7 (7.5)		7 (5.7)		0.598	
PT: Chills	1 (1.1)	6 (6.5)	3 (2.5)	4 (3.3)	0.457	0.274
Injection site redness	3 (3.2)		4 (3.3)		0.983	
PT: Application site redness	2 (2.2)	2 (2.2)	2 (1.6)	2 (1.6)	0.784	0.784

Adverse reactions (PT MedDRA term)	Humoral immunogenicity				[a]p-value	
	Antibody titres below median* (n= 93)		Antibody titres above median* (n= 122)			
	After any dose		After any dose			
	After 1st dose	After 2nd dose	After 1st dose	After 2nd dose		
SOC: *Musculoskeletal and connective tissue disorders*						
Arm pain<br>*PT:*<br>*Pain in extremity*	17 (18.3)		36 (29.5)		0.058	
	**10 (10.8)**	9 (9.7)	**26 (21.3)**	13 (10.7)	**0.040**	0.815
Muscle pain<br>*PT:*<br>*Myalgia*	17 (18.3)		24 (19.7)		0.797	
	2 (2.2)	15 (16.1)	7 (5.7)	19 (15.6)	0.193	0.912
Joint pain<br>*PT:*<br>*Arthralgia*	10 (10.8)		8 (6.6)		0.271	
	0 (0)	10 (10.8)	1 (0.8)	7 (5.7)	0.382	0.177
Shoulder pain<br>*PT:*<br>*Musculoskeletal pain*	5 (5.4)		4 (3.3)		0.447	
	4 (4.3)	1 (1.1)	2 (1.6)	1 (0.8)	0.240	0.847
SOC: *Injury, poisoning and procedural complications*						
Injection site swelling<br>*PT:*<br>*Application site swelling*	5 (5.4)		10 (8.2)		0.421	
	3 (3.2)	2 (2.2)	9 (7.4)	2 (1.6)	0.189	0.784
SOC: Vaccination site pruritus						
Injection site pruritus<br>*PT:*<br>*Vaccination site pruritus*	1 (1.1)		2 (1.6)		0.727	
	0 (0)	1 (1.1)	0 (0)	2 (1.6)	NA	0.727

Adverse reactions (PT MedDRA term)	Humoral immunogenicity				[a]p-value	
	Antibody titres below median* (n= 93)		Antibody titres above median* (n= 122)			
	After any dose		After any dose			
	After 1st dose	After 2nd dose	After 1st dose	After 2nd dose		
SOC: Nervous system disorders						
Headache *PT: Headache*	29 (31.2)		30 (24.6)		0.283	
	13 (14.0)	19 (20.4)	14 (11.5)	17 (13.9)	0.583	0.206
Facial paralysis *PT: Facial paralysis*	0 (0)		0 (0)		NA	
	0 (0)	0 (0)	0 (0)	0 (0)	NA	NA
Insomnia *PT: Insomnia*	2 (2.2)		2 (1.6)		0.784	
	0 (0)	2 (2.2)	0 (0)	2 (1.6)	NA	0.784
SOC: Gastrointestinal disorders						
Nausea *PT: Nausea*	2 (2.2)		5 (4.1)		0.425	
	1 (1.1)	1 (1.1)	4 (3.3)	1 (0.8)	0.288	0.847
Diarrhea *PT: Diarrhoea*	1 (1.1)		2 (1.6)		0.727	
	1 (1.1)	0 (0)	1 (0.8)	1 (0.8)	0.847	0.382
Vomiting *PT: Vomiting*	0 (0)		0 (0)		NA	
	0 (0)	0 (0)	0 (0)	0 (0)	NA	NA

Adverse reactions (PT MedDRA term)	Humoral immunogenicity				[a]p-value	
	Antibody titres below median* (n= 93)		Antibody titres above median* (n= 122)			
	After any dose		After any dose			
	After 1st dose	After 2nd dose	After 1st dose	After 2nd dose		
**Unsolicited adverse reactions**						
SOC: *Musculoskeletal and connective tissue disorders*						
Other musculoskeletal disorders	4 (4.3)		3 (2.5)		0.451	
PT: Musculoskeletal disorder	1 (1.1)	3 (3.2)	0 (0)	3 (2.5)	0.251	0.735
SOC: *Skin and subcutaneous tissue disorders*						
Petechia, ecchymosis	0 (0)		0 (0)		NA	
PT: *Ecchymosis*	0 (0)	0 (0)	0 (0)	0 (0)	NA	NA
SOC: *Nervous system disorders*						
Cognitive alteration	0 (0)		0 (0)		NA	
PT: Cognitive disorder	0 (0)	0 (0)	0 (0)	0 (0)	NA	NA
Alterations of smell and taste	0 (0)		1 (0.8)		0.382	
PT: Parosmia	0 (0)	0 (0)	0 (0)	1 (0.8)	NA	0.382
Paresthesia and hyperesthesia	1 (1.1)		2 (1.6)		0.727	
PT: Dysaesthesia	0 (0)	1 (1.1)	2 (1.6)	0 (0)	0.215	0.251
Presyncope, syncope and vasovagal syncope	0 (0)		0 (0)		NA	
PT: Syncope	0 (0)	0 (0)	0 (0)	0 (0)	NA	NA

Adverse reactions (PT MedDRA term)	Humoral immunogenicity				[a]p-value	
	Antibody titres below median* (n= 93)		Antibody titres above median* (n= 122)			
	After any dose		After any dose			
	After 1st dose	After 2nd dose	After 1st dose	After 2nd dose		
Sleepiness, hypersomnia *PT: Hypersomnia*	0 (0)		0 (0)		NA	
	0 (0)	0 (0)	0 (0)	0 (0)	NA	NA
Instability sensation, vertigo, sickness *PT: Dizziness*	1 (1.1)		2 (1.6)		0.727	
	0 (0)	1 (1.1)	1 (0.8)	1 (0.8)	0.382	0.847
Tremor *PT: Tremor*	0 (0)		0 (0)		NA	
	0 (0)	0 (0)	0 (0)	0 (0)	NA	NA
SOC: *Gastrointestinal disorders*						
Gastrointestinal disorders *PT: Gastrointestinal disorder*	1 (1.1)		0 (0)		0.251	
	1 (1.1)	0 (0)	0 (0)	0 (0)	0.251	NA
SOC: *Respiratory, thoracic and mediastinal disorders*						
Asthma *PT: Asthma*	0 (0)		0 (0)		NA	
	0 (0)	0 (0)	0 (0)	0 (0)	NA	NA
Rhinitis, nasal discharge *PT: Rhinitis*	0 (0)		1 (0.8)		0.382	
	0 (0)	0 (0)	0 (0)	1 (0.8)	NA	0.382

Adverse reactions (PT MedDRA term)	Humoral immunogenicity				[a]p-value	
	Antibody titres below median* (n= 93)		Antibody titres above median* (n= 122)			
	After any dose		After any dose			
	After 1st dose	After 2nd dose	After 1st dose	After 2nd dose		
SOC: *Investigations*						
Hypertension, hypotension *PT: Blood pressure abnormal*	2 (2.2)		0 (0)		0.104	
	0 (0)	2 (2.2)	0 (0)	0 (0)	NA	0.104
SOC: *General disorders and administration site conditions*						
Influenza-like symptoms PT: *Influenza like illness*	0 (0)		0 (0)		NA	
	0 (0)	0 (0)	0 (0)	0 (0)	NA	NA
Sensation of heat, sensation of cold PT: *Temperature regulation disorder*	1 (1.1)		1 (0.8)		0.847	
	1 (1.1)	0 (0)	0 (0)	1 (0.8)	0.251	0.382
Chest pain PT: *Chest pain*	0 (0)		0 (0)		NA	
	0 (0)	0 (0)	0 (0)	0 (0)	NA	NA
Hiporexia, anorexia PT: *Decreased appetite*	0 (0)		0 (0)		NA	
	0 (0)	0 (0)	0 (0)	0 (0)	NA	NA
Inflammation in extremities other than the vaccinated arm PT: *Inflammation*	0 (0)		0 (0)		NA	
	0 (0)	0 (0)	0 (0)	0 (0)	NA	NA

Adverse reactions (PT MedDRA term)	Humoral immunogenicity				[a]p-value	
	Antibody titres below median* (n= 93)		Antibody titres above median* (n= 122)			
	After any dose		After any dose			
	After 1st dose	After 2nd dose	After 1st dose	After 2nd dose		
SOC: *Ear and labyrinth disorders*						
PT: *Ear pain*	0 (0)		0 (0)		NA	
	0 (0)	0 (0)	0 (0)	0 (0)	NA	NA
SOC: *Skin and subcutaneous tissue disorders*						
General pruritus PT: *Pruritus*	2 (2.2)		0 (0)		0.104	
	1 (1.1)	1 (1.1)	0 (0)	0 (0)	0.251	0.251
Sweat PT: *Cold sweat*	0 (0)		0 (0)		NA	
	0 (0)	0 (0)	0 (0)	0 (0)	NA	NA
SOC: *Immune system disorders*						
Immediate hypersensitivity, delayed hypersensitivity, exanthema, urticaria, rash PT: *Hypersensitivity*	1 (1.1)		1 (0.8)		0.847	
	0 (0)	1 (1.1)	1 (0.8)	0 (0)	0.382	0.251
SOC: *Cardiac disorders*						
Tachycardia PT: *Tachycardia*	0 (0)		1 (0.8)		0.382	
	0 (0)	0 (0)	0 (0)	1 (0.8)	NA	0.382

Adverse reactions (PT MedDRA term)	Humoral immunogenicity				[a]p-value	
	Antibody titres below median* (n= 93)		Antibody titres above median* (n= 122)			
	After any dose		After any dose			
	After 1st dose	After 2nd dose	After 1st dose	After 2nd dose		
SOC: *Blood and lymphatic system disorders*						
Lymphadenopathy	**0 (0)**		**6 (4.9)**		**0.030**	
PT: *Lymphadenopathy*	0 (0)	**0 (0)**	1 (0.8)	**5 (4.1)**	0.382	**0.048**
SOC: *Infections and infestations*						
Herpetic infection	0 (0)		0 (0)		NA	
PT: *Herpes virus infection*	0 (0)	0 (0)	0 (0)	0 (0)	NA	NA

Distribution of proportions of severe solicited and unsolicited AR are displayed by elicited quantitative humoral immunogenicity and dose; [a]chi-square test; AR: adverse reaction; SOC: System Organ Class; PT: Preferred term; NA: not applicable, p-value not calculable because of absence of valid values in both comparison groups; in bold type: statistically significant results. Results are provided as absolute and relative (%) numbers for each variable, *Median value of antibody titres (P25, P75)= 7.69 (0.73,10.00) on a semiquantitative scale ranging from 0 to >10

Table S6 Assessment of antibody titres (antibody titres above-below median): severe solicited AR

Adverse reactions (PT MedDRA term)	Humoral immunogenicity				[a]p-value	
	Humoral immunogenicity		Humoral immunogenicity			
	Antibody titres below median* (n= 93)		Antibody titres above median* (n= 122)			
	After any dose		After any dose			
	After 1st dose	After 2nd dose	After 1st dose	After 2nd dose		
**Solicited adverse reactions**						
SOC: *General disorders and administration site conditions*						
Injection site pain<br>*PT: Injection site pain*	6 (6.5)		12 (9.8)		0.375	
	6 (6.5)	0 (0)	10 (8.2)	3 (2.5)	0.629	0.128
Fatigue/astenia<br>*PT:*<br>*Fatigue*	**13 (14.0)**		**6 (4.9)**		**0.020**	
	3 (3.2)	10 (10.8)	1 (0.8)	5 (4.1)	0.196	0.058
Fever<br>*PT:*<br>*Pyrexia*	**0 (0)**		**7 (5.7)**		**0.019**	
	0 (0)	**0 (0)**	3 (2.5)	**5 (4.1)**	0.128	**0.048**
Malaise<br>*PT:*<br>*Malaise*	4 (4.3)		12 (9.8)		0.126	
	0 (0)	4 (4.3)	1 (0.8)	10 (8.2)	0.382	0.251
Injection site redness<br>*PT:*<br>*Application site redness*	0 (0)		1 (0.8)		0.382	
	0 (0)	0 (0)	1 (0.8)	0 (0)	0.382	NA
SOC: *Musculoskeletal and connective tissue disorders*						

Adverse reactions (PT MedDRA term)	Humoral immunogenicity				[a]p-value	
	Humoral immunogenicity		Humoral immunogenicity			
	Antibody titres below median* (n= 93)		Antibody titres above median* (n= 122)			
	After any dose		After any dose			
	After 1st dose	After 2nd dose	After 1st dose	After 2nd dose		
Arm pain<br>*PT:*<br>*Pain in extremity*	8 (8.6)		15 (12.3)		0.385	
	6 (6.5)	2 (2.2)	10 (8.2)	5 (4.1)	0.629	0.425
Muscle pain<br>*PT:*<br>*Myalgia*	7 (7.5)		11 (9.0)		0.696	
	0 (0)	7 (7.5)	4 (3.3)	8 (6.6)	0.078	0.782
Joint pain<br>*PT:*<br>*Arthralgia*	8 (8.6)		5 (4.1)		0.170	
	0 (0)	8 (8.6)	0 (0)	5 (4.1)	NA	0.170
Shoulder pain<br>*PT:*<br>*Musculoskeletal pain*	1 (1.1)		1 (0.8)		0.847	
	1 (1.1)	0 (0)	0 (0)	1 (0.8)	0.251	0.251
SOC: *Injury, poisoning and procedural complications*						
Injection site swelling<br>*PT:*<br>*Application site swelling*	0 (0)		4 (3.3)		0.078	
	0 (0)	0 (0)	4 (3.3)	0 (0)	0.078	NA
SOC: Vaccination site pruritus						
Injection site pruritus<br>*PT:*<br>*Vaccination site pruritus*	0 (0)		0 (0)		NA	
	0 (0)	0 (0)	0 (0)	0 (0)	NA	NA
SOC: Nervous system disorders						
Headache	11 (11.8)		12 (9.8)		0.640	

Adverse reactions (PT MedDRA term)	Humoral immunogenicity				[a]p-value	
	Humoral immunogenicity		Humoral immunogenicity			
	Antibody titres below median* (n= 93)		Antibody titres above median* (n= 122)			
	After any dose		After any dose			
	After 1st dose	After 2nd dose	After 1st dose	After 2nd dose		
PT: Headache	3 (3.2)	9 (9.7)	5 (4.1)	7 (5.7)	0.738	0.276

Distribution of proportions of severe solicited and unsolicited AR are displayed by elicited qualitative humoral immunogenicity and dose; [a]chi-square test; AR: adverse reaction; SOC: System Organ Class; PT: Preferred term; NA: not applicable, p-value not calculable because of absence of valid values in both comparison groups; in bold type: statistically significant results. Results are provided as absolute and relative (%) numbers for each variable, * Median value of antibody titres (P25, P75)= 7.69 (0.73,10.00) on a semiquantitative scale ranging from 0 to >10

Figure S1: Summary of results of immunogenicity and reactogenicity of the main population

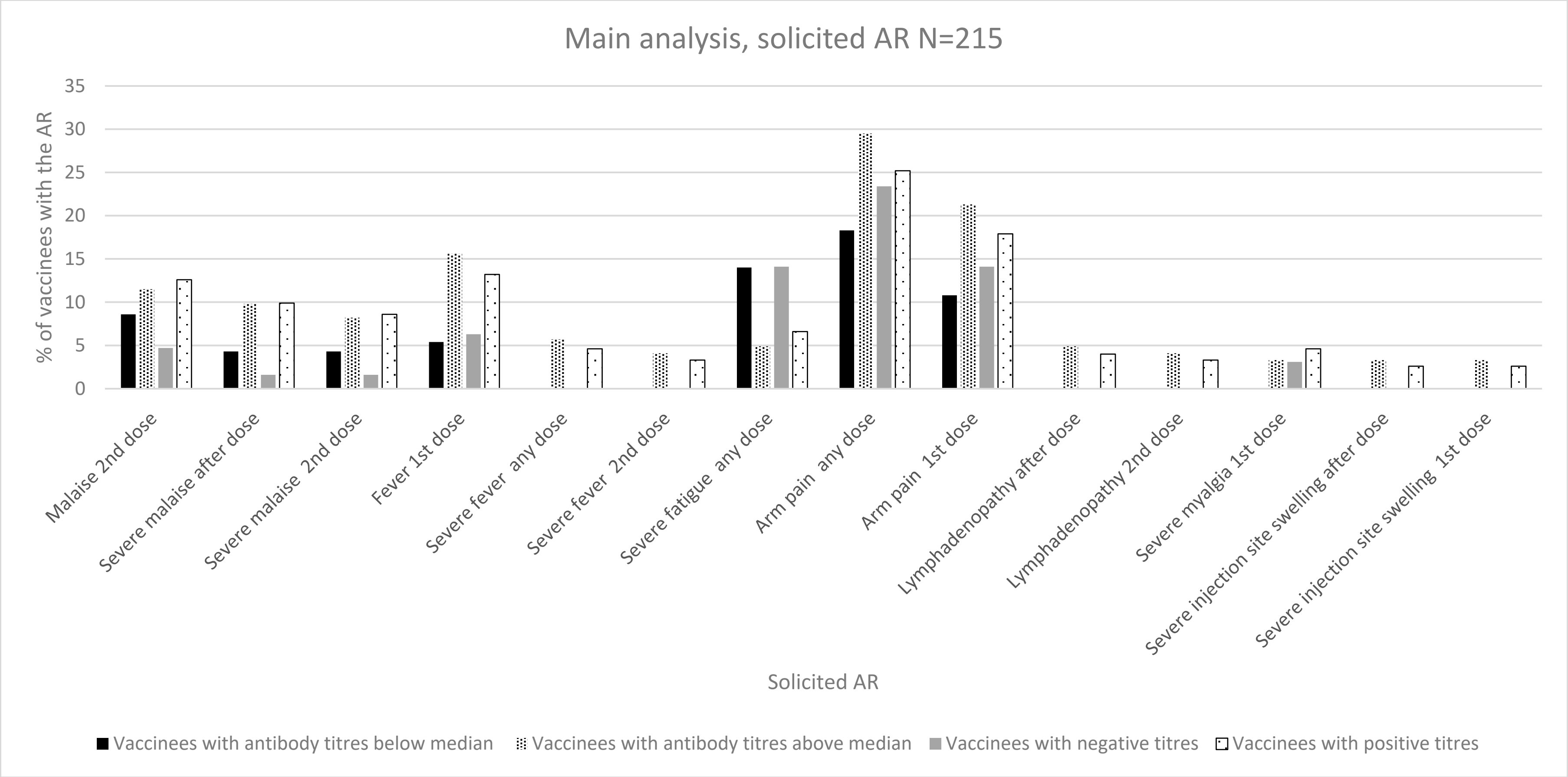

Bar diagram of results of main analysis. Immunogenicity is expressed as antibody positiveness (positive-negative titres) and antibody titres (antibody titres below-above median) data (%). Reactogenicity results are represented as % of vaccinees developing each solicited AR

ii.ii Sensitivity analysis 1

Table S7: Characteristics of subpopulation 1

Covariates	Total population (N= 3563)
**Age**	
N	3563
Mean (SD)	41.84 (12.79)
Median (P25, P75)	42.00 (30.00, 53.00)
min, max	18.00, 81.00
**Gender**	
Male (n, %)	976 (27.4)
Female (n, %)	2587 (72.6)
**Occupational SARS-CoV-2 contact**	
In contact with SARS-CoV-2 patients	1501 (42.1)
Without contact with SARS-CoV-2 patients	1957 (54.9)
**[a]Previous SARS-CoV-2 infection**	
Yes	444 (12.5)
No	3119 (87.5)
**Comorbidities**	
[b]Any comorbidity	1305 (36.6)
Arterial hypertension	141 (4.0)
Diabetes mellitus	53 (1.5)
Heart failure	13 (0.4)
Chronic bronchitis	12 (0.3)
Asthma	126 (3.5)
Rheumatic/immune-mediated disease	33 (0.9)
**Drug allergies**	302 (8.5)
**Food allergies**	146 (4.1)
**Type of administered vaccine**	
BNT16b2	
First dose:	2698 (75.7)
Second dose:	2167 (60.8)
mRNA-1273	
First dose:	865 (24.3)
Second dose:	773 (21.7)
**[c]Population type**	
HCP	3460
SOTR	103
**Humoral immunogenicity (semiquantitative ab titres)**	
Positive	3487 (97.9)
Negative	76 (2.1)
**IgG titres**	326
Positive	322 (98.8)
Negative	4 (1.2)
**Cellular immunogenicity**	59
Positive	51 (86)
Negative	8 (14)

Covariables of studied population. [a]Diagnosis by RCP, antigen test and antibody positive serology; [b]Number of vaccinees with at least one comorbidity; [c]HCPs: Healthcare professionals; SOTR: solid-organ transplant recipients. Results are provided as absolute and relative (%) numbers for each variable

Table S8: Assessment of antibody positiveness (positive-negative titres)

Variable	Antibody positiveness											
	**Negative antibody titres (n= 76)**						**Positive antibody titres (n= 3487)**					
	**After 1st vaccine dose**	**p-value**	**After 2nd vaccine dose**	**p-value**	**After any dose**	**p-value**	**After 1st vaccine dose**	**p-value**	**After 2nd vaccine dose**	**p-value**	**After any dose**	**p-value**
**Proportions of AR**												
Development of any AR (n, %)	56 (73.7)	0.074	45 (59.2)	0.156	64 (84.2)	0.810	2222 (63.7)	0.074	2335 (67.0)	0.156	2971 (85.2)	0.810
Development of any mild AR (n, %)	21 (27.6)	0.063	17 (22.4)	0.308	29 (38.2)	0.198	667 (19.1)	0.063	622 (17.8)	0.308	1089 (31.2)	0.198
Development of any moderate AR (n, %)	26 (34.2)	0.950	27 (35.5)	0.113	42 (55.3)	0.321	1181 (33.9)	0.950	1557 (44.7)	0.113	2123 (60.9)	0.321
Development of any severe AR (n, %)	17 (22.4)	0.520	**13 (17.1)**	**0.017**	25 (32.9)	0.207	677 (19.4)	0.520	**1036 (29.7)**	**0.017**	1397 (40.1)	0.207
**Maximum intensity**												
Grade 1 (n, %)	18 (33.3)	0.164	**12 (27.3)**	**0.003**	**15 (23.8)**	**0.020**	480 (22.9)	0.164	**261 (11.6)**	**0.003**	**350 (12.2)**	**0.020**
Grade 2 (n, %)	19 (35.2)		**19 (43.2)**		**23 (36.5)**		943 (44.9)		**949 (42.3)**		**1132 (39.3)**	
Grade 3 (n, %)	17 (31.5)		**13 (29.5)**		**25 (39.7)**		677 (32.2)		**1036 (46.1)**		**1397 (48.5)**	
Likert score												
Mean (SD)	4.96 (2.41)	0.241	**4.95 (2.37)**	**0.001**	**5.48 (2.47)**	**0.031**	5.34 (2.46)	0.241	**6.10 (2.06)**	**0.004**	**6.15 (2.36)**	**0.031**

Antibody titres according to proportions (rate) of AR and intensity of AR. AR: adverse reaction; Ab: antibody; in bold type: statistically significant results. Results are provided as absolute and relative (%) numbers for each variable

Table S9: Assessment of antibody positiveness (positive-negative titres): solicited and unsolicited AR

Adverse reactions (PT MedDRA term)	Humoral immunogenicity				[a]p-value	
	Negative antibody titres		Positive antibody titres			
	After any dose		After any dose			
**Solicited adverse reactions**						
SOC: *General disorders and administration site conditions*						
Injection site pain	35 (46.1)		1348 (38.7)		0.190	
PT: Injection site pain	30 (39.5)	13 (17.1)	1058 (30.3)	603 (17.3)	0.090	0.970
Fatigue/astenia	17 (22.4)		988 (28.3)		0.253	
PT: Fatigue	7 (9.2)	11 (14.5)	223 (14.6)	782 (22.4)	0.940	0.100
Fever	17 (22.4)		1095 (31.4)		0.090	
PT: Pyrexia	**9 (11.8)**	**11 (14.5)**	**216 (6.2)**	**974 (27.9)**	**0.050**	**0.010**
Malaise	**7 (9.2)**		**622 (17.8)**		**0.050**	
PT: Malaise	3 (3.9)	**4 (5.3)**	138 (4.0)	**517 (14.8)**	0.990	**0.020**
Chills	5 (6.6)		448 (12.8)		0.100	
PT: Chills	3 (3.9)	**2 (2.6)**	83 (2.4)	**385 (11.0)**	0.380	**0.020**
Injection site redness	2 (2.6)		155 (4.4)		0.450	
PT: Application site redness	1 (1.3)	1 (1.3)	90 (2.6)	72 (2.1)	0.490	0.650
SOC: *Musculoskeletal and connective tissue disorders*						
	24 (31.6)		1048 (30.1)		0.770	

Adverse reactions (PT MedDRA term)	Humoral immunogenicity				[a]p-value	
	Negative antibody titres		Positive antibody titres			
	After any dose		After any dose			
Arm pain<br>*PT:*<br>*Pain in extremity*	17 (22.4)	13 (17.1)	690 (19.8)	548 (15.7)	0.580	0.740
Muscle pain<br>*PT:*<br>*Myalgia*	**5 (6.6)**		**709 (20.3)**		**0.003**	
	2 (2.6)	**4 (5.3)**	117 (3.4)	**621 (17.8)**	0.730	**0.004**
Joint pain<br>*PT:*<br>*Arthralgia*	4 (5.3)		328 (9.4)		0.220	
	0 (0)	4 (5.3)	32 (0.9)	307 (8.8)	0.400	0.280
Shoulder pain<br>*PT:*<br>*Musculoskeletal pain*	**8 (10.5)**		**106 (3.0)**		**0.002**	
	1 (1.3)	**7 (9.2)**	61 (1.7)	**52 (1.5)**	0.770	**<0.001**
SOC: *Injury, poisoning and procedural complications*						
Injection site swelling<br>*PT:*<br>*Application site swelling*	5 (6.6)		246 (7.1)		0.870	
	5 (6.6)	0 (0)	144 (4.1)	124 (3.6)	0.290	0.090
SOC: Vaccination site pruritus						
Injection site pruritus<br>*PT:*<br>*Vaccination site pruritus*	0 (0)		73 (2.1)		0.200	
	0 (0)	0 (0)	40 (1.1)	39 (1.1)	0.350	0.350
SOC: Nervous system disorders						
Headache<br>*PT:*<br>*Headache*	**11 (14.5)**		**967 (27.7)**		**0.010**	
	5 (6.6)	**8 (10.5)**	330 (9.5)	**750 (21.5)**	0.390	**0.020**

Adverse reactions (PT MedDRA term)	Humoral immunogenicity				[a]p-value	
	Negative antibody titres		Positive antibody titres			
	After any dose		After any dose			
Facial paralysis	0 (0)		1 (0)		0.880	
PT: Facial paralysis	0 (0)	0 (0)	1 (0)	0 (0)	0.880	NA
Insomnia	0 (0)		35 (1.0)		0.380	
PT: Insomnia	0 (0)	0 (0)	9 (0.3)	26 (0.7)	0.660	0.450
SOC: Gastrointestinal disorders						
Nausea	2 (2.6)		164 (4.7)		0.400	
PT: Nausea	0 (0)	2 (2.6)	53 (1.5)	119 (3.4)	0.280	0.710
Diarrhea	3 (3.9)		107 (3.1)		0.660	
PT: Diarrhoea	1 (1.3)	2 (2.6)	35 (1.0)	73 (2.1)	0.790	0.750
Vomiting	2 (2.6)		61 (1.7)		0.560	
PT: Vomiting	0 (0)	2 (2.6)	15 (0.4)	50 (1.4)	0.570	0.390
**Unsolicited adverse reactions**						
SOC: *Musculoskeletal and connective tissue disorders*						
Other musculoskeletal disorders	0 (0)		90 (2.6)		0.160	
PT: Musculoskeletal disorder	0 (0)	0 (0)	32 (0.9)	62 (1.8)	0.400	0.240
SOC: *Skin and subcutaneous tissue disorders*						
Petechia, ecchymosis	0 (0)		14 (0.4)		0.580	

Adverse reactions (PT MedDRA term)	Humoral immunogenicity				[a]p-value	
	**Negative antibody titres**		**Positive antibody titres**			
	**After any dose**		**After any dose**			
PT: *Ecchymosis*	0 (0)	0 (0)	10 (0.3)	4 (0.1)	0.640	0.770
SOC: *Nervous system disorders*						
Cognitive alteration	**1 (1.3)**		**5 (0.1)**		**0.010**	
PT: Cognitive disorder	**1 (1.3)**	0 (0)	**3 (0.1)**	2 (0.1)	**0.002**	0.830
Alterations of smell and taste	0 (0)		8 (0.2)		0.680	
PT: Parosmia	0 (0)	0 (0)	5 (0.1)	3 (0.1)	0.740	0.800
Paresthesia and hyperesthesia	0 (0)		27 (0.8)		0.440	
PT: Dysaesthesia	0 (0)	0 (0)	13 (0.4)	16 (0.5)	0.590	0.550
Presyncope, syncope and vasovagal syncope	0 (0)		7 (0.2)		0.700	
PT: Syncope	0 (0)	0 (0)	2 (0.1)	5 (0.1)	0.830	0.740
Sleepiness, hypersomnia	0 (0)		16 (0.5)		0.550	
PT: Hypersomnia	0 (0)	0 (0)	3 (0.1)	13 (0.4)	0.800	0.590
Instability sensation, vertigo, sickness	4 (5.3)		86 (2.5)		0.120	
PT: Dizziness	0 (0)	**4 (5.3)**	30 (0.9)	**58 (1.7)**	0.420	**0.020**
Tremor	0 (0)		7 (0.2)		0.70	

**Adverse reactions (PT MedDRA term)**	**Humoral immunogenicity**				**[a]p-value**	
	**Negative antibody titres**		**Positive antibody titres**			
	**After any dose**		**After any dose**			
PT:<br>*Tremor*	0 (0)	0 (0)	2 (0.1)	5 (0.1)	0.830	0.740
SOC: *Gastrointestinal disorders*						
Gastrointestinal disorders<br>*PT:*<br>*Gastrointestinal disorder*	1 (1.3)		35 (1.0)		0.790	
	0 (0)	1 (1.3)	12 (0.3)	22 (0.6)	0.610	0.460
SOC: *Respiratory, thoracic and mediastinal disorders*						
Asthma<br>*PT:*<br>*Asthma*	0 (0)		4 (0.1)		0.770	
	0 (0)	0 (0)	1 (0)	2 (0.1)	0.880	0.830
Rhinitis, nasal discharge<br>*PT:*<br>*Rhinitis*	0 (0)		26 (0.7)		0.450	
	0 (0)	0 (0)	11 (0.3)	16 (0.5)	0.620	0.550
SOC: *Investigations*						
Hypertension, hypotension<br>*PT:*<br>*Blood pressure abnormal*	1 (1.3)		12 (0.3)		0.160	
	0 (0)	**1 (1.3)**	4 (0.1)	**7 (0.2)**	0.770	**0.040**
SOC: *General disorders and administration site conditions*						

Adverse reactions (PT MedDRA term)	Humoral immunogenicity				[a]p-value	
	Negative antibody titres		Positive antibody titres			
	After any dose		After any dose			
Influenza-like symptoms PT: *Influenza like illness*	0 (0)		47 (1.3)		0.310	
	0 (0)	0 (0)	16 (0.5)	32 (0.9)	0.550	0.400
Sensation of heat, sensation of cold PT: *Temperature regulation disorder*	**1 (1.3)**		**5 (0.1)**		**0.010**	
	**1 (1.3)**	0 (0)	**2 (0.1)**	5 (0.1)	**0.002**	0.740
Chest pain PT: *Chest pain*	0 (0)		8 (0.2)		0.680	
	0 (0)	0 (0)	0 (0)	8 (0.2)	NA	0.68
Hiporexia, anorexia PT: *Decreased appetite*	0 (0)		4 (0.1)		0.770	
	0 (0)	0 (0)	0 (0)	4 (0.1)	NA	0.77
Inflammation in extremities other than the vaccinated arm PT: *Inflammation*	0 (0)		11 (0.3)		0.620	
	0 (0)	0 (0)	5 (0.1)	7 (0.2)	0.740	0.700
SOC: *Ear and labyrinth disorders*						
PT: *Ear pain*	0 (0)		3 (0.1)		0.800	
	0 (0)	0 (0)	1 (0)	2 (0.1)	0.880	0.830
SOC: *Skin and subcutaneous tissue disorders*						

**Adverse reactions (PT MedDRA term)**	**Humoral immunogenicity**				**[a]p-value**	
	**Negative antibody titres**		**Positive antibody titres**			
	**After any dose**		**After any dose**			
General pruritus PT: *Pruritus*	**1 (1.3)**		**7 (0.2)**		**0.040**	
	0 (0)	**1 (1.3)**	4 (0.1)	**3 (0.1)**	0.770	**0.002**
Sweat PT: *Cold sweat*	0 (0)		16 (0.5)		0.550	
	0 (0)	0 (0)	2 (0.1)	14 (0.4)	0.830	0.580
SOC: *Immune system disorders*						
Immediate hypersensitivity, delayed hypersensitivity, exanthema, urticaria, rash PT: *Hypersensitivity*	1 (1.3)		49 (1.4)		0.950	
	0 (0)	1 (1.3)	24 (0.7)	31 (0.9)	0.470	0.700
SOC: *Cardiac disorders*						
Tachycardia PT: *Tachycardia*	0 (0)		11 (0.3)		0.620	
	0 (0)	0 (0)	5 (0.1)	6 (0.2)	0.740	0.720
SOC: *Blood and lymphatic system disorders*						
Lymphadenopathy PT: *Lymphadenopathy*	4 (5.3)		142 (4.1)		0.600	
	2 (2.6)	2 (2.6)	39 (1.1)	107 (3.1)	0.220	0.830
SOC: *Infections and infestations*						

Adverse reactions (PT MedDRA term)	Humoral immunogenicity				[a]p-value	
	Negative antibody titres		Positive antibody titres			
	After any dose		After any dose			
Herpetic infection	0 (0)		10 (0.3)		0.640	
PT: *Herpes virus infection*	0 (0)	0 (0)	6 (0.2)	4 (0.1)	0.720	0.770

Distribution of proportions of solicited and unsolicited AR are displayed by elicited immunogenicity (humoral and cellular) and dose; [a]chi-square test; AR: adverse reaction; SOC: System Organ Class; PT: Preferred term; NA: not applicable, p-value not calculable because of absence of valid values in both comparison groups; in bold type: statistically significant results. Results are provided as absolute and relative (%) numbers for each variable

Table S10: Assessment of antibody positiveness (positive-negative titres): severe solicited AR

Severe adverse reactions (PT MedDRA term)	Humoral immunogenicity				[a]p-value	
	Negative antibody titres		Positive antibody titres			
	After any dose		After any dose			
	After 1st dose	After 2nd dose	After 1st dose	After 2nd dose		
**Solicited adverse reactions**						
SOC: *General disorders and administration site conditions*						
Injection site pain<br>*PT: Injection site pain*	11 (14.5)		338 (9.7)		0.170	
	**10 (13.2)**	1 (1.3)	**229 (6.6)**	138 (4.0)	**0.020**	0.240
Fatigue/astenia<br>*PT:*<br>*Fatigue*	7 (9.2)		483 (13.9)		0.250	
	1 (1.3)	6 (7.9)	114 (3.3)	389 (11.2)	0.340	0.370
Fever<br>*PT:*<br>*Pyrexia*	2 (2.6)		110 (3.2)		0.800	
	0 (0)	2 (2.6)	19 (0.5)	92 (2.6)	0.520	0.990
Malaise<br>*PT:*<br>*Malaise*	**1 (1.3)**		**305 (8.7)**		**0.020**	
	0 (0)	**1 (1.3)**	53 (1.5)	**258 (7.4)**	0.280	**0.040**
Injection site redness<br>*PT:*<br>*Application site redness*	1 (1.3)		39 (1.1)		0.870	
	0 (0)	1 (1.3)	21 (0.6)	20 (0.6)	0.500	0.400
SOC: *Musculoskeletal and connective tissue disorders*						
Arm pain<br>*PT:*<br>*Pain in extremity*	5 (6.6)		392 (11.2)		0.200	
	4 (5.3)	1 (1.3)	238 (6.8)	187 (5.4)	0.590	0.120

Severe adverse reactions (PT MedDRA term)	Humoral immunogenicity				[a]p-value	
	Negative antibody titres		Positive antibody titres			
	After any dose		After any dose			
	After 1st dose	After 2nd dose	After 1st dose	After 2nd dose		
Muscle pain *PT: Myalgia*	3 (3.9)		333 (9.5)		0.100	
	1 (1.3)	2 (2.6)	45 (1.3)	296 (8.5)	0.980	0.070
Joint pain *PT: Arthralgia*	2 (2.6)		184 (5.3)		0.310	
	0 (0)	2 (2.6)	17 (0.5)	171 (4.9)	0.540	0.360
Shoulder pain *PT: Musculoskeletal pain*	1 (1.3)		28 (0.8)		0.620	
	0 (0)	1 (1.3)	11 (0.3)	16 (0.5)	0.620	0.280
SOC: *Injury, poisoning and procedural complications*						
Injection site swelling *PT: Application site swelling*	1 (1.3)		70 (2.0)		0.670	
	1 (1.3)	0 (0)	43 (1.2)	33 (0.9)	0.950	0.390
SOC: Vaccination site pruritus						
Injection site pruritus *PT: Vaccination site pruritus*	0 (0)		18 (0.5)		0.530	
	0 (0)	0 (0)	8 (0.2)	11 (0.3)	0.680	0.620

Severe adverse reactions (PT MedDRA term)	Humoral immunogenicity				[a]p-value	
	Negative antibody titres		Positive antibody titres			
	After any dose		After any dose			
	After 1st dose	After 2nd dose	After 1st dose	After 2nd dose		
SOC: Nervous system disorders						
Headache *PT: Headache*	**3 (3.9)**		**419 (12.0)**		**0.030**	
	1 (1.3)	**2 (2.6)**	115 (3.3)	**331 (9.5)**	0.340	**0.040**

Distribution of proportions of severe solicited and unsolicited AR are displayed by elicited immunogenicity (humoral and cellular) and dose; [a]chi-square test; AR: adverse reaction; SOC: System Organ Class; PT: Preferred term; NA: not applicable, p-value not calculable because of absence of valid values in both comparison groups; in bold type: statistically significant results. Results are provided as absolute and relative (%) numbers for each variable

Table S11: Assessment of antibody titres (antibody titres above-below median):

Variable	Antibody titres below median* (n= 440)						Antibody titres above median * (n= 3123)					
	After 1st vaccine dose	p-value	After 2nd vaccine dose	p-value	After any dose	p-value	After 1st vaccine dose	p-value	After 2nd vaccine dose	p-value	After any dose	p-value
**Proportions of AR**												
Development of any AR (n, %)	295 (67.0)	0.147	291 (66.1)	0.753	382 (86.8)	0.302	1983 (63.5)	0.147	2089 (66.9)	0.753	2653 (85.0)	0.302
Development of any mild AR (n, %)	**102 (23.2)**	**0.028**	**99 (22.5)**	**0.008**	**168 (38.2)**	**0.001**	**586 (18.8)**	**0.028**	**540 (17.3)**	**0.008**	**950 (30.4)**	**0.001**
Development of any moderate AR (n, %)	147 (33.4)	0.825	188 (42.7)	0.436	257 (58.4)	0.280	1060 (33.9)	0.825	1396 (44.7)	0.436	1908 (61.1)	0.280
Development of any severe AR (n, %)	90 (20.5)	0.581	**108 (24.5)**	**0.016**	163 (37.0)	0.190	604 (19.3)	0.581	**941 (30.1)**	**0.016**	1259 (40.3)	0.190
**Maximum intensity**												
Grade 1 (n, %)	75 (26.9)	0.223	**47 (17.0)**	**0.006**	**62 (16.9)**	**0.017**	423 (22.6)	0.223	**226 (11.2)**	**0.006**	**303 (11.8)**	**0.017**
Grade 2 (n, %)	114 (40.9)		**121 (43.8)**		**142 (38.7)**		848 (45.2)		**847 (42.1)**		**1013 (39.3)**	
Grade 3 (n, %)	90 (32.3)		**108 (39.1)**		**163 (44.4)**		604 (32.2)		**941 (46.7)**		**1259 (48.9)**	
Likert score Mean (SD)	5.27 (2.21)	0.685	**5.64 (2.14)**	**0.003**	5.91 (2.17)	0.067	5.34 (2.50)	0.685	**6.14 (2.05)**	**0.003**	6.16 (2.38)	0.067

Antibody titres (in median titres </> median) according to proportions (rate) of AR and intensity of AR. AR: adverse reaction; Ab: antibody; in bold type: statistically significant results. Results are provided as absolute and relative (%) numbers for each variable, * Median value of antibody titres (P25, P75)= 10.00 (10.00, 10.00) on a semiquantitative scale ranging from 0 to >10

Table S12: Assessment of antibody titres (antibody titres above-below median): solicited and unsolicited AR

Adverse reactions (PT MedDRA term)	Development of any AR				[a]p-value	
	Antibody titres below median* (n=440)		Antibody titres below median* (n=3123)			
	After any dose		After any dose			
	After 1st dose	After 2nd dose	After 1st dose	After 2nd dose		
**Solicited adverse reactions**						
SOC: *General disorders and administration site conditions*						
Injection site pain	**192 (43.6)**		**1191 (38.1)**		**0.030**	
PT: Injection site pain	145 (33.0)	82 (18.6)	943 (30.2)	534 (17.1)	0.240	0.420
Fatigue/astenia	112 (25.5)		893 (28.6)		0.170	
PT: Fatigue	41 (9.3)	85 (19.3)	279 (8.9)	708 (22.7)	0.790	0,110
Fever	139 (31.6)		973 (31.2)		0.850	
PT: Pyrexia	37 (8.4)	114 (25.9)	188 (6.0)	871 (27.9)	0.050	0.380
Malaise	68 (15.5)		561 (18.0)		0.200	
PT: Malaise	16 (3.6)	56 (12.7)	125 (4.0)	465 (14.9)	0.710	0.230
Chills	45 (10.2)		408 (13.1)		0.090	
PT: Chills	10 (2.3)	**35 (8.0)**	76 (2.4)	**352 (11.3)**	0.840	**0.040**
Injection site redness	17 (3.9)		140 (4.5)		0.550	
PT: Application site redness	10 (2.3)	8 (1.8)	81 (2.6)	65 (2.1)	0.700	0.720

Adverse reactions (PT MedDRA term)	Development of any AR				[a]p-value	
	Antibody titres below median* (n=440)		Antibody titres below median* (n=3123)			
	After any dose		After any dose			
	After 1st dose	After 2nd dose	After 1st dose	After 2nd dose		
SOC: *Musculoskeletal and connective tissue disorders*						
Arm pain *PT: Pain in extremity*	126 (28.6)		946 (30.3)		0.480	
	84 (19.1)	69 (15.7)	623 (19.9)	492 (15.8)	0.670	0.970
Muscle pain *PT: Myalgia*	94 (21.4)		620 (19.9)		0.460	
	17 (3.9)	79 (18.0)	102 (3.3)	546 (17.5)	0.510	0.810
Joint pain *PT: Arthralgia*	43 (9.8)		289 (9.3)		0.730	
	5 (1.1)	38 (8.6)	27 (0.9)	273 (8.7)	0.570	0.940
Shoulder pain *PT: Musculoskeletal pain*	**21 (4.8)**		**93 (3.0)**		**0.040**	
	8 (1.8)	**13 (3.0)**	54 (1.7)	**46 (1.5)**	0.890	**0.020**
SOC: *Injury, poisoning and procedural complications*						
Injection site swelling *PT: Application site swelling*	37 (8.4)		214 (6.9)		0.230	
	25 (5.7)	14 (3.2)	124 (4.0)	110 (3.5)	0.090	0.720

Adverse reactions (PT MedDRA term)	Development of any AR				[a]p-value	
	Antibody titres below median* (n=440)		Antibody titres below median* (n=3123)			
	After any dose		After any dose			
	After 1st dose	After 2nd dose	After 1st dose	After 2nd dose		
SOC: Vaccination site pruritus						
Injection site pruritus *PT: Vaccination site pruritus*	11 (2.5)		62 (2.0)		0.480	
	6 (1.4)	5 (1.1)	34 (1.1)	34 (1.1)	0.610	0.930
SOC: Nervous system disorders						
Headache *PT: Headache*	118 (26.8)		860 (27.5)		0.750	
	38 (8.6)	89 (20.2)	297 (9.5)	669 (21.4)	0.560	0.570
Facial paralysis *PT: Facial paralysis*	0 (0)		1 (0)		0.710	
	0 (0)	0 (0)	1 (0)	0 (0)	0.710	NA
Insomnia *PT: Insomnia*	7 (1.6)		28 (0.9)		0.170	
	1 (0.2)	6 (1.4)	8 (0.3)	20 (0.6)	0.910	0.100
SOC: Gastrointestinal disorders						
Nausea *PT: Nausea*	25 (5.7)		141 (4.5)		0.280	
	**13 (3.0)**	12 (2.7)	**40 (1.3)**	109 (3.5)	**0.010**	0.410

Adverse reactions (PT MedDRA term)	Development of any AR				[a]p-value	
	Antibody titres below median* (n=440)		Antibody titres below median* (n=3123)			
	After any dose		After any dose			
	After 1st dose	After 2nd dose	After 1st dose	After 2nd dose		
Diarrhea *PT: Diarrhoea*	13 (3.0)		97 (3.1)		0.860	
	3 (0.7)	10 (2.3)	33 (1.1)	65 (2.1)	0.460	0.790
Vomiting *PT: Vomiting*	8 (1.8)		55 (1.8)		0.930	
	1 (0.2)	7 (1.6)	14 (0.4)	45 (1.4)	0.500	0.810
**Unsolicited adverse reactions**						
SOC: *Musculoskeletal and connective tissue disorders*						
Other musculoskeletal disorders *PT: Musculoskeletal disorder*	10 (2.3)		80 (2.6)		0.720	
	3 (0.7)	7 (1.6)	29 (0.9)	55 (1.8)	0.610	0.800
SOC: *Skin and subcutaneous tissue disorders*						
Petechia, ecchymosis PT: *Ecchymosis*	1 (0.2)		13 (0.4)		0.560	
	1 (0.2)	0 (0)	9 (0.3)	4 (0.1)	0.820	0.450
SOC: *Nervous system disorders*						
Cognitive alteration *PT: Cognitive disorder*	1 (0.2)		5 (0.2)		0.750	
	1 (0.2)	0 (0)	3 (0.1)	2 (0.1)	0.440	0.600

Adverse reactions (PT MedDRA term)	Development of any AR				^a p-value	
	Antibody titres below median* (n=440)		Antibody titres below median* (n=3123)			
	After any dose		After any dose			
	After 1st dose	After 2nd dose	After 1st dose	After 2nd dose		
Alterations of smell and taste	2 (0.5)		6 (0.2)		0.280	
PT: *Parosmia*	1 (0.2)	1 (0.2)	4 (0.1)	2 (0.1)	0.600	0.270
Paresthesia and hyperesthesia	5 (1.1)		22 (0.7)		0.330	
PT: *Dysaesthesia*	2 (0.5)	3 (0.7)	11 (0.4)	13 (0.4)	0.740	0.440
Presyncope, syncope and vasovagal syncope	1 (0.2)		6 (0.2)		0.880	
PT: *Syncope*	0 (0)	1 (0.2)	2 (0.1)	4 (0.1)	0.560	0.600
Sleepiness, hypersomnia	0 (0)		16 (0.5)		0.130	
PT: *Hypersomnia*	0 (0)	0 (0)	3 (0.1)	13 (0.4)	0.520	0.180
Instability sensation, vertigo, sickness	8 (1.8)		82 (2.6)		0.310	
PT: *Dizziness*	2 (0.5)	7 (1.6)	28 (0.9)	55 (1.8)	0.340	0.800
Tremor	0 (0)		7 (0.2)		0.320	
PT: *Tremor*	0 (0)	0 (0)	2 (0.1)	5 (0.1)	0.600	0.400
SOC: *Gastrointestinal disorders*						

Adverse reactions (PT MedDRA term)	Development of any AR				^a p-value	
	**Antibody titres below median* (n=440)**		**Antibody titres below median* (n=3123)**			
	**After any dose**		**After any dose**			
	**After 1st dose**	**After 2nd dose**	**After 1st dose**	**After 2nd dose**		
Gastrointestinal disorders *PT: Gastrointestinal disorder*	7 (1.6)		29 (0.9)		0.190	
	1 (0.2)	5 (1.1)	11 (0.4)	18 (0.6)	0.670	0.170
SOC: *Respiratory, thoracic and mediastinal disorders*						
Asthma *PT: Asthma*	1 (0.2)		3 (0.1)		0.440	
	**1 (0.2)**	0 (0)	**0 (0)**	2 (0.1)	**0.010**	0.600
Rhinitis, nasal discharge *PT: Rhinitis*	2 (0.5)		24 (0.8)		0.47	
	2 (0.5)	0 (0)	9 (0.3)	16 (0.5)	0.560	0.130
SOC: *Investigations*						

Adverse reactions (PT MedDRA term)	Development of any AR				[a]p-value	
	Antibody titres below median* (n=440)		Antibody titres below median* (n=3123)			
	After any dose		After any dose			
	After 1st dose	After 2nd dose	After 1st dose	After 2nd dose		
Hypertension, hypotension<br>*PT:*<br>*Blood pressure abnormal*	3 (0.7)		10 (0.3)		0.240	
	0 (0)	**3 (0.7)**	4 (0.1)	**5 (0.2)**	0.450	**0.030**
SOC: *General disorders and administration site conditions*						
Influenza-like symptoms<br>PT:<br>*Influenza like illness*	4 (0.9)		43 (1.4)		0.420	
	2 (0.5)	3 (0.7)	14 (0.4)	29 (0.9)	0.990	0.610
Sensation of heat, sensation of cold<br>PT: *Temperature regulation disorder*	3 (0.7)		10 (0.3)		0.240	
	0 (0)	**3 (0.7)**	4 (0.1)	**5 (0.2)**	0.450	**0.030**
Chest pain<br>PT:<br>*Chest pain*	0 (0)		8 (0.3)		0.290	
	0 (0)	0 (0)	0 (0)	8 (0.3)	NA	0.290
Hiporexia, anorexia<br>PT:<br>*Decreased appetite*	0 (0)		4 (0.1)		0.450	
	0 (0)	0 (0)	0 (0)	4 (0.1)	NA	0.450
	2 (0.5)		9 (0.3)		0.560	

Adverse reactions (PT MedDRA term)	Development of any AR				[a]p-value	
	Antibody titres below median* (n=440)		Antibody titres below median* (n=3123)			
	After any dose		After any dose			
	After 1st dose	After 2nd dose	After 1st dose	After 2nd dose		
Inflammation in extremities other than the vaccinated arm PT: *Inflammation*	0 (0)	2 (0.5)	5 (0.2)	5 (0.2)	0.400	0.190
SOC: *Ear and labyrinth disorders*						
PT: *Ear pain*	0 (0)		3 (0.1)		0.520	
	0 (0)	0 (0)	1 (0)	2 (0.1)	0.710	0.600
SOC: *Skin and subcutaneous tissue disorders*						
General pruritus PT: *Pruritus*	2 (0.5)		6 (0.2)		0.280	
	1 (0.2)	1 (0.2)	3 (0.1)	3 (0.1)	0.440	0.440
Sweat PT: *Cold sweat*	2 (0.5)		14 (0.4)		0.990	
	0 (0)	2 (0.5)	2 (0.1)	12 (0.4)	0.600	0.830
SOC: *Immune system disorders*						
Immediate hypersensitivity, delayed hypersensitivity, exanthema, urticaria, rash PT: *Hypersensitivity*	8 (1.8)		42 (1.3)		0.430	
	1 (0.2)	7 (1.6)	23 (0.7)	25 (0.8)	0.220	0.100

Adverse reactions (PT MedDRA term)	Development of any AR				[a]p-value	
	Antibody titres below median* (n=440)		Antibody titres below median* (n=3123)			
	After any dose		After any dose			
	After 1st dose	After 2nd dose	After 1st dose	After 2nd dose		
SOC: *Cardiac disorders*						
Tachycardia<br>PT:<br>*Tachycardia*	0 (0)		11 (0.4)		0.210	
	0 (0)	0 (0)	5 (0.2)	6 (0.2)	0.400	0.360
SOC: *Blood and lymphatic system disorders*						
Lymphadenopathy<br>PT:<br>*Lymphadenopathy*	20 (4.5)		126 (4.0)		0.610	
	7 (1.6)	14 (3.2)	34 (1.1)	95 (3.0)	0.360	0.870
SOC: *Infections and infestations*						
Herpetic infection<br>PT:<br>*Herpes virus infection*	1 (0.2)		9 (0.3)		0.820	
	1 (0.2)	0 (0)	5 (0.2)	4 (0.1)	0.750	0.450

Distribution of proportions of non-severe solicited and unsolicited AR are displayed by elicited humoral immunogenicity (mean Ab titres </> Q2) and dose; [a]chi-square test; AR: adverse reaction; SOC: System Organ Class; PT: Preferred term; NA: not applicable; in bold type: statistically significant results. Results are provided as absolute and relative (%) numbers for each variable, *Median value of antibody titres (P25, P75)= 10.00 (10.00, 10.00) on a semiquantitative scale ranging from 0 to >10

Table S13: Assessment of antibody titres (antibody titres above-below median): severe solicited AR

Adverse reactions (PT MedDRA term)	Development of any severe AR				[a]p-value	
	Antibody titres below median* (n=440)		Antibody titres above median* (n=3123)			
	After any dose		After any dose			
	After 1st dose	After 2nd dose	After 1st dose	After 2nd dose		
**Solicited adverse reactions**						
SOC: *General disorders and administration site conditions*						
Injection site pain	47 (10.7)		302 (9.7)		0.500	
PT: Injection site pain	33 (7.5)	14 (3.2)	206 (6.6)	125 (4.0)	0.480	0.410
Fatigue/astenia	49 (11.1)		441 (14.1)		0.090	
PT: Fatigue	11 (2.5)	40 (9.1)	104 (3.3)	355 (11.4)	0.360	0.150
Fever	10 (2.3)		102 (3.3)		0.260	
PT: Pyrexia	2 (0.5)	8 (1.8)	17 (0.5)	86 (2.8)	0.810	0.250
Malaise	32 (7.3)		274 (8.8)		0.290	
PT: Malaise	7 (1.6)	25 (5.7)	46 (1.5)	234 (7.5)	0.850	0.170
Chills	NA		NA		NA	
PT: Chills	NA	NA	NA	NA	NA	NA
Injection site redness	6 (1.4)		34 (1.1)		0.610	
PT: Application site redness	4 (0.9)	2 (0.5)	17 (0.5)	19 (0.6)	0.350	0.690

Adverse reactions (PT MedDRA term)	Development of any severe AR				[a]p-value	
	Antibody titres below median* (n=440)		Antibody titres above median* (n=3123)			
	After any dose		After any dose			
	After 1st dose	After 2nd dose	After 1st dose	After 2nd dose		
SOC: *Musculoskeletal and connective tissue disorders*						
Arm pain<br>*PT:*<br>*Pain in extremity*	43 (9.8)		354 (11.3)		0.330	
	30 (6.8)	15 (3.4)	212 (6.8)	173 (5.5)	0.980	0.060
Muscle pain<br>*PT:*<br>*Myalgia*	35 (8.0)		301 (9.6)		0.260	
	5 (1.1)	29 (6.6)	41 (1.3)	269 (8.6)	0.760	0.150
Joint pain<br>*PT:*<br>*Arthralgia*	25 (5.7)		161 (5.2)		0.640	
	3 (0.7)	22 (5.0)	14 (0.4)	151 (4.8)	0.510	0.880
Shoulder pain<br>*PT:*<br>*Musculoskeletal pain*	3 (0.7)		26 (0.8)		0.740	
	2 (0.5)	1 (0.2)	9 (0.3)	16 (0.5)	0.560	0.420
SOC: *Injury, poisoning and procedural complications*						
Injection site swelling<br>*PT:*<br>*Application site swelling*	12 (2.7)		59 (1.9)		0.240	
	**10 (2.3)**	2 (0.5)	**34 (1.1)**	31 (1.0)	**0.030**	0.270

Adverse reactions (PT MedDRA term)	Development of any severe AR				[a]p-value	
	Antibody titres below median* (n=440)		Antibody titres above median* (n=3123)			
	After any dose		After any dose			
	After 1st dose	After 2nd dose	After 1st dose	After 2nd dose		
SOC: Vaccination site pruritus						
Injection site pruritus	0 (0)		18 (0.6)		0.110	
PT: Vaccination site pruritus	0 (0)	0 (0)	8 (0.3)	11 (0.4)	0.290	0.210
SOC: Nervous system disorders						
Headache	**39 (8.9)**		**383 (12.3)**		**0.040**	
PT: Headache	11 (2.5)	**28 (6.4)**	105 (3.4)	**305 (9.8)**	0.340	**0.020**

Distribution of proportions of severe solicited and unsolicited AR are displayed by elicited humoral immunogenicity (mean Ab titres </> Q2) and dose; [a]chi-square test; AR: adverse reaction; SOC: System Organ Class; PT: Preferred term; NA: not applicable; in bold type: statistically significant results. Results are provided as absolute and relative (%) numbers for each variable, *Median value of antibody titres (P25, P75)= 10.00 (10.00, 10.00) on a semiquantitative scale ranging from 0 to >10

Table S14: Cellular immunogenicity assessment

Variable	Negative cellular immunogenicity (n= 8)						Positive cellular immunogenicity (n= 51)					
	**After 1st vaccine dose**	**p-value**	**After 2nd vaccine dose**	**p-value**	**After any dose**	**p-value**	**After 1st vaccine dose**	**p-value**	**After 2nd vaccine dose**	**p-value**	**After any dose**	**p-value**
**Proportions of AR**												
Development of any AR (n, %)	6 (75.0)	0.7159	6 (75.0)	0.7159	6 (75.0)	0.1355	35 (68.6)	0.7159	35 (68.6)	0.7159	47 (92.2)	0.1355
Development of any mild AR (n, %)	1 (12.5)	0.8157	3 (37.5)	0.1946	4 (50.0)	0.2466	8 (15.7)	0.8157	9 (17.6)	0.1946	15 (29.4)	0.2466
Development of any moderate AR (n, %)	3 (37.5)	0.9894	4 (50.0)	0.7162	5 (62.5)	0.9894	19 (37.3)	0.9894	22 (43.1)	0.7162	32 (62.7)	0.9894
Development of any severe AR (n, %)	3 (37.5)	0.2563	1 (12.5)	0.1691	3 (37.5)	0.4783	10 (19.6)	0.2563	19 (37.3)	0.1691	26 (51.0)	0.4783
**Maximum intensity**												
Grade 1 (n, %)	1 (16.7)	0.6394	1 (20.0)	0.3423	1 (16.7)	0.9067	7 (21.2)	0.6394	5 (14.3)	0.3423	5 (10.9)	0.9067
Grade 2 (n, %)	2 (33.3)		3 (60.0)		2 (33.3)		16 (48.5)		11 (31.4)		15 (32.6)	
Grade 3 (n, %)	3 (50.0)		1 (20.0)		3 (50.0)		10 (30.3)		19 (54.3)		26 (56.5)	
Likert score Mean (SD)	5.67 (1.97)	0.755	5.20 (2.28)	0.256	5.48 (2.47)	0.467	5.32 (1.89)	0.755	6.32 (1.85)	0.256	6.45 (1.73)	0.467

Cellular immunogenicity according to proportions (rate) of AR and intensity of AR. AR: adverse reaction; Ab: antibody; in bold type: statistically significant results. Results are provided as absolute and relative (%) numbers for each variable

Table S15: Cellular immunogenicity assessment: solicited and unsolicited AR

Adverse reactions (PT MedDRA term)	Cellular immunogenicity				[a]p-value	
	Negative		Positive			
	After any dose		After any dose			
	After 1st dose	After 2nd dose	After 1st dose	After 2nd dose		
**Solicited adverse reactions**						
SOC: *General disorders and administration site conditions*						
Injection site pain<br>*PT: Injection site pain*	1 (12.5)		23 (45.1)		0.080	
	0 (0)	1 (12.5)	17 (33.3)	14 (27.5)	0.050	0.370
Fatigue/astenia<br>*PT:*<br>*Fatigue*	3 (37.5)		14 (27.5)		0.560	
	2 (25.0)	3 (37.5)	3 (5.9)	12 (23.5)	0.070	0.400
Fever<br>*PT:*<br>*Pyrexia*	2 (25.0)		17 (33.3)		0.639	
	1 (12.5)	2 (25.0)	5 (9.8)	13 (25.5)	0.810	0.980
Malaise<br>*PT:*<br>*Malaise*	2 (25.0)		10 (19.6)		0.720	
	0 (0)	2 (25.0)	2 (3.9)	9 (17.6)	0.570	0.620
Chills<br>*PT:*<br>*Chills*	1 (12.5)		7 (13.7)		0.925	
	1 (12.5)	0 (0)	5 (9.8)	2 (3.9)	0.810	0.570
Injection site redness<br>*PT:*<br>*Application site redness*	0 (0)		2 (3.9)		0.570	
	0 (0)	0 (0)	1 (2.0)	1 (2.0)	0.690	0.690

Adverse reactions (PT MedDRA term)	Cellular immunogenicity				[a]p-value	
	Negative		Positive			
	After any dose		After any dose			
	After 1st dose	After 2nd dose	After 1st dose	After 2nd dose		
SOC: *Musculoskeletal and connective tissue disorders*						
Arm pain *PT: Pain in extremity*	3 (37.5)		15 (29.4)		0.640	
	3 (37.5)	1 (12.5)	8 (15.7)	7 (13.7)	0.140	0.930
Muscle pain *PT: Myalgia*	1 (12.5)		13 (25.5)		0.420	
	1 (12.5)	0 (0)	2 (3.9)	12 (23.5)	0.300	0.120
Joint pain *PT: Arthralgia*	0 (0)		7 (13.7)		0.260	
	0 (0)	0 (0)	0 (0)	7 (13.7)	NA	0.260
Shoulder pain *PT: Musculoskeletal pain*	1 (12.5)		1 (2.0)		0.130	
	**1 (12.5)**	0 (0)	**0 (0)**	1 (2.0)	**0.010**	0.700
SOC: *Injury, poisoning and procedural complications*						
Injection site swelling *PT: Application site swelling*	0 (0)		4 (7.8)		0.410	
	0 (0)	0 (0)	3 (5.9)	2 (3.9)	0.480	0.570

Adverse reactions (PT MedDRA term)	Cellular immunogenicity				[a]p-value	
	Negative		Positive			
	After any dose		After any dose			
	After 1st dose	After 2nd dose	After 1st dose	After 2nd dose		
SOC: Vaccination site pruritus						
Injection site pruritus	0 (0)		0 (0)		NA	
PT: Vaccination site pruritus	0 (0)	0 (0)	0 (0)	0 (0)	NA	NA
SOC: Nervous system disorders						
Headache	3 (37.5)		16 (31.4)		0.730	
PT: Headache	2 (25.0)	2 (25.0)	6 (11.8)	12 (23.5)	0.310	0.930
Facial paralysis	0 (0)		0 (0)		NA	
PT: Facial paralysis	0 (0)	0 (0)	0 (0)	0 (0)	NA	NA
Insomnia	0 (0)		0 (0)		NA	
PT: Insomnia	0 (0)	0 (0)	0 (0)	0 (0)	NA	NA
SOC: Gastrointestinal disorders						
Nausea	0 (0)		3 (5.9)		0.480	
PT: Nausea	0 (0)	0 (0)	1 (2.0)	2 (3.9)	0.700	0.600
Diarrhea	1 (12.5)		0 (0)		0.010	
PT: Diarrhoea	1 (12.5)	78 (2.2)	0 (0)	0 (0)	0.010	NA
Vomiting	0 (0)		0 (0)		NA	
PT: Vomiting	0 (0)	0 (0)	0 (0)	0 (0)	NA	NA

Adverse reactions (PT MedDRA term)	Cellular immunogenicity				[a]p-value	
	Negative		Positive			
	After any dose		After any dose			
	After 1st dose	After 2nd dose	After 1st dose	After 2nd dose		
**Unsolicited adverse reactions**						
SOC: *Musculoskeletal and connective tissue disorders*						
Other musculoskeletal disorders *PT: Musculoskeletal disorder*	1 (12.5)		1 (2.0)		0.130	
	0 (0)	1 (12.5)	0 (0)	1 (2.0)	NA	0.130
SOC: *Skin and subcutaneous tissue disorders*						
Petechia, ecchymosis PT: *Ecchymosis*	0 (0)		0 (0)		NA	
	0 (0)	0 (0)	0 (0)	0 (0)	NA	NA
SOC: *Nervous system disorders*						
Cognitive alteration *PT: Cognitive disorder*	0 (0)		0 (0)		NA	
	0 (0)	0 (0)	0 (0)	0 (0)	NA	NA
Alterations of smell and taste *PT: Parosmia*	0 (0)		0 (0)		NA	
	0 (0)	0 (0)	0 (0)	0 (0)	NA	NA
Paresthesia and hyperesthesia *PT: Dysaesthesia*	0 (0)		0 (0)		NA	
	0 (0)	0 (0)	0 (0)	0 (0)	NA	NA

**Adverse reactions (PT MedDRA term)**	**Cellular immunogenicity**				**[a]p-value**	
	**Negative**		**Positive**			
	**After any dose**		**After any dose**			
	**After 1st dose**	**After 2nd dose**	**After 1st dose**	**After 2nd dose**		
Presyncope, syncope and vasovagal syncope *PT: Syncope*	0 (0)		0 (0)		NA	
	0 (0)	0 (0)	0 (0)	0 (0)	NA	NA
Sleepiness, hypersomnia *PT: Hypersomnia*	0 (0)		0 (0)		NA	
	0 (0)	0 (0)	0 (0)	0 (0)	NA	NA
Instability sensation, vertigo, sickness *PT: Dizziness*	0 (0)		2 (3.9)		0.57	
	0 (0)	0 (0)	1 (2.0)	1 (2.0)	0.700	0.700
Tremor *PT: Tremor*	0 (0)		0 (0)		NA	
	0 (0)	0 (0)	0 (0)	0 (0)	NA	NA
SOC: *Gastrointestinal disorders*						
Gastrointestinal disorders *PT: Gastrointestinal disorder*	**1 (12.5)**		**0 (0)**		**0.010**	
	**1 (12.5)**	25 (0.7)	**0 (0)**	0 (0)	**0.010**	NA
SOC: *Respiratory, thoracic and mediastinal disorders*						
Asthma *PT: Asthma*	0 (0)		0 (0)		NA	
	0 (0)	0 (0)	0 (0)	0 (0)	NA	NA

Adverse reactions (PT MedDRA term)	Cellular immunogenicity				[a]p-value	
	Negative		Positive			
	After any dose		After any dose			
	After 1st dose	After 2nd dose	After 1st dose	After 2nd dose		
Rhinitis, nasal discharge<br>*PT:*<br>*Rhinitis*	0 (0)		0 (0)		NA	
	0 (0)	0 (0)	0 (0)	0 (0)	NA	NA
SOC: *Investigations*						
Hypertension, hypotension<br>*PT:*<br>*Blood pressure abnormal*	0 (0)		0 (0)		NA	
	0 (0)	0 (0)	0 (0)	0 (0)	NA	NA
SOC: *General disorders and administration site conditions*						
Influenza-like symptoms<br>PT:<br>*Influenza like illness*	0 (0)		1 (2.0)		0.700	
	0 (0)	0 (0)	1 (2.0)	0 (0)	0.700	NA
Sensation of heat, sensation of cold<br>PT: *Temperature regulation disorder*	0 (0)		0 (0)		NA	
	0 (0)	0 (0)	0 (0)	0 (0)	NA	NA
Chest pain<br>PT:<br>*Chest pain*	0 (0)		0 (0)		NA	
	0 (0)	0 (0)	0 (0)	0 (0)	NA	NA
Hiporexia, anorexia<br>PT:<br>*Decreased appetite*	0 (0)		0 (0)		NA	
	0 (0)	0 (0)	0 (0)	0 (0)	NA	NA

Adverse reactions (PT MedDRA term)	Cellular immunogenicity				[a]p-value	
	Negative		Positive			
	After any dose		After any dose			
	After 1st dose	After 2nd dose	After 1st dose	After 2nd dose		
Inflammation in extremities other than the vaccinated arm PT: *Inflammation*	0 (0)		0 (0)		NA	
	0 (0)	0 (0)	0 (0)	0 (0)	NA	NA
SOC: *Ear and labyrinth disorders*						
PT: *Ear pain*	0 (0)		0 (0)		NA	
	0 (0)	0 (0)	0 (0)	0 (0)	NA	NA
SOC: *Skin and subcutaneous tissue disorders*						
General pruritus PT: *Pruritus*	0 (0)		0 (0)		NA	
	0 (0)	0 (0)	0 (0)	0 (0)	NA	NA
Sweat PT: *Cold sweat*	0 (0)		0 (0)		NA	
	0 (0)	0 (0)	0 (0)	0 (0)	NA	NA
SOC: *Immune system disorders*						
Immediate hypersensitivity, delayed hypersensitivity, exanthema, urticaria, rash PT: *Hypersensitivity*	0 (0)		0 (0)		NA	
	0 (0)	0 (0)	0 (0)	0 (0)	NA	NA

Adverse reactions (PT MedDRA term)	Cellular immunogenicity				ᵃp-value	
	Negative		Positive			
	After any dose		After any dose			
	After 1st dose	After 2nd dose	After 1st dose	After 2nd dose		
SOC: *Cardiac disorders*						
Tachycardia	0 (0)		0 (0)		NA	
PT: *Tachycardia*	0 (0)	0 (0)	0 (0)	0 (0)	NA	NA
SOC: *Blood and lymphatic system disorders*						
Lymphadenopathy	0 (0)		2 (3.9)		0.570	
PT: *Lymphadenopathy*	0 (0)	0 (0)	1 (2.0)	1 (2.0)	0.700	0.700
SOC: *Infections and infestations*						
Herpetic infection	0 (0)		1 (2.0)		0.700	
PT: *Herpes virus infection*	0 (0)	0 (0)	1 (2.0)	0 (0)	0.700	NA

Distribution of proportions of non-severe solicited and unsolicited AR are displayed by elicited cellular immunogenicity and dose; [a]chi-square test; AR: adverse reaction; SOC: System Organ Class; PT: Preferred term; NA: not applicable, p-value not calculable because of absence of valid values in both comparison groups; in bold type: statistically significant. Results are provided as absolute and relative (%) numbers for each variable

Table S16: Cellular immunogenicity assessment: severe solicited AR

Severe adverse reactions (PT MedDRA term)	Cellular immunogenicity				[a]p-value	
	Negative		Positive			
	After any dose		After any dose			
	After 1st dose	After 2nd dose	After 1st dose	After 2nd dose		
**Solicited adverse reactions**						
SOC: *General disorders and administration site conditions*						
Injection site pain	0 (0)		7 (13.7)		0.260	
PT: Injection site pain	0 (0)	0 (0)	2 (3.9)	5 (9.8)	0,570	0.350
Fatigue/astenia	1 (12.5)		10 (19.6)		0.630	
PT: Fatigue	1 (12.5)	0 (0)	2 (3.9)	9 (17.6)	0.300	0.200
Fever	0 (0)		0 (0)		NA	
PT: Pyrexia	0 (0)	0 (0)	0 (0)	0 (0)		
Malaise	0 (0)		6 (11.8)		0.310	
PT: Malaise	0 (0)	0 (0)	0 (0)	6 (11.8)	NA	0.310
Injection site redness	0 (0)		1 (2.0)		0.700	
PT: Application site redness	0 (0)	0 (0)	1 (2.0)	0 (0)	0.70	NA
SOC: *Musculoskeletal and connective tissue disorders*						

Severe adverse reactions (PT MedDRA term)	Cellular immunogenicity				[a]p-value	
	Negative		Positive			
	After any dose		After any dose			
	After 1st dose	After 2nd dose	After 1st dose	After 2nd dose		
Arm pain<br>*PT:*<br>*Pain in extremity*	2 (25.0)		5 (9.8)		0.220	
	2 (25.0)	0 (0)	4 (7.8)	1 (2.0)	0.140	0.700
Muscle pain<br>*PT:*<br>*Myalgia*	0 (0)		6 (11.8)		0.310	
	0 (0)	0 (0)	1 (2.0)	5 (9.8)	0.700	0.350
Joint pain<br>*PT:*<br>*Arthralgia*	0 (0)		5 (9.8)		0.350	
	0 (0)	0 (0)	0 (0)	5 (9.8)	NA	0.350
Shoulder pain<br>*PT:*<br>*Musculoskeletal pain*	0 (0)		0 (0)		NA	
	0 (0)	0 (0)	0 (0)	0 (0)	NA	NA
SOC: *Injury, poisoning and procedural complications*						

Severe adverse reactions (PT MedDRA term)	Cellular immunogenicity				[a]p-value	
	Negative		Positive			
	After any dose		After any dose			
	After 1st dose	After 2nd dose	After 1st dose	After 2nd dose		
Injection site swelling<br>*PT:*<br>*Application site swelling*	0 (0)		2 (3.9)		0.570	
	0 (0)	0 (0)	2 (3.9)	0 (0)	0.570	NA
SOC: Vaccination site pruritus						
Injection site pruritus<br>*PT:*<br>*Vaccination site pruritus*	0 (0)		0 (0)		NA	
	0 (0)	0 (0)	0 (0)	0 (0)	NA	NA
SOC: Nervous system disorders						
Headache<br>*PT:*<br>*Headache*	1 (12.5)		8 (15.7)		0.820	
	0 (0)	1 (12.5)	1 (2.0)	7 (13.7)	0.700	0.930

Distribution of proportions of severe solicited and unsolicited AR are displayed by elicited cellular immunogenicity and dose; [a]chi-square test; AR: adverse reaction; SOC: System Organ Class; PT: Preferred term; NA: not applicable, p-value not calculable because of absence of valid values in both comparison groups; in bold type: statistically significant. Results are provided as absolute and relative (%) numbers for each variable

Figure S2: Summary of results of immunogenicity and reactogenicity of sensitivity analysis 1

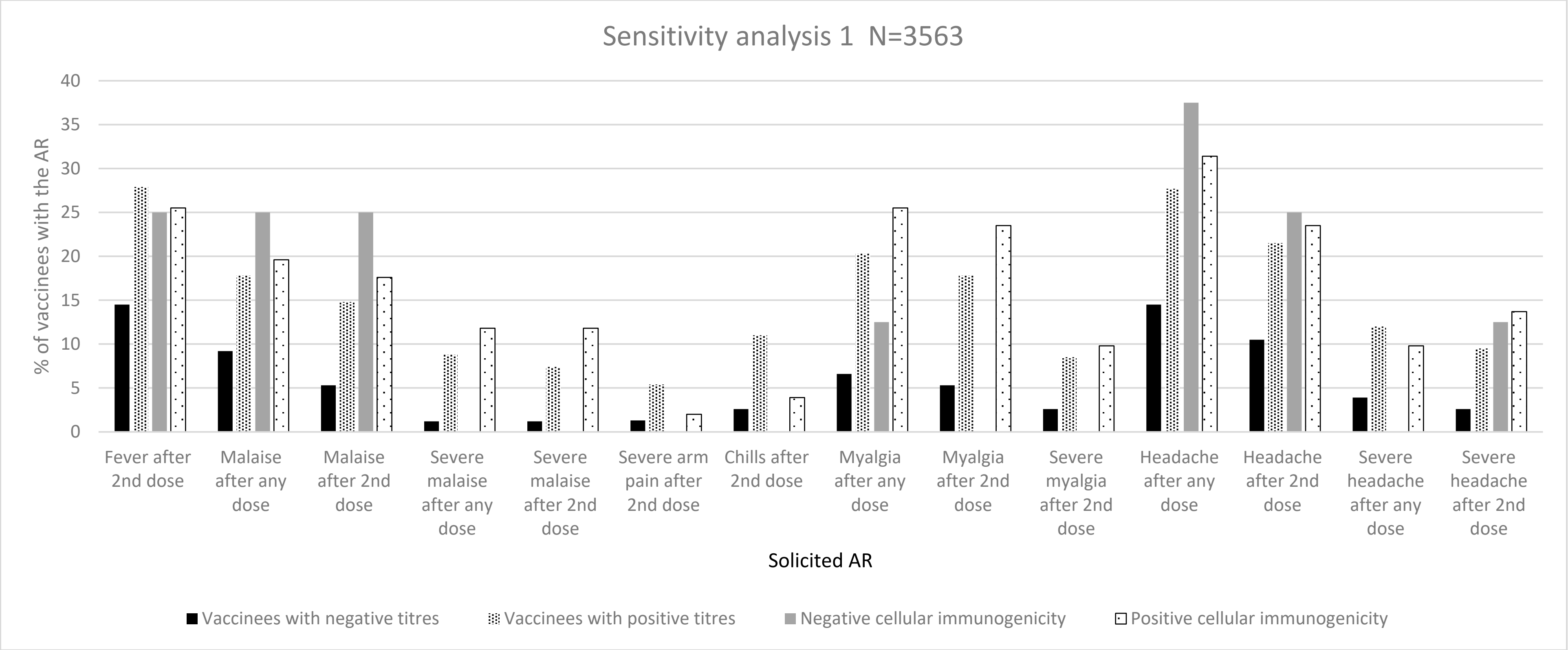

Bar diagram of results of main analysis. Immunogenicity is expressed as antibody positiveness (positive-negative titres) and cellular immunogenicity (positive-negative) data (%). Reactogenicity results are represented as % of vaccinees developing each solicited AR

ii.iii Sensitivity analysis 2

Table S17: Characteristics of subpopulation 2

Covariates	Total population (N=597)
**Age**	
N	3563
Mean (SD)	39.42 (12.69)
Median (P25, P75)	38.00 (28.00 , 50.00)
min, max	18.00 , 69.00
**Gender**	
Male (n, %)	173 (29.0)
Female (n, %)	424 (71.0)
**Occupational SARS-CoV-2 contact**	
In contact with SARS-CoV-2 patients	282 (47.2)
Without contact with SARS-CoV-2 patients	315 (52.8)
**[a]Previous SARS-CoV-2 infection**	
Yes	269 (45.1)
No	328 (54.9)
**Comorbidities**	
[b]Any comorbidity	212 (35.5)
Arterial hypertension	25 (4.2)
Diabetes mellitus	9 (1.5)
Heart failure	2 (0.3)
Chronic bronchitis	2 (0.3)
Asthma	19 (3.2)
Rheumatic/immune-mediated disease	6 (1.0)
**Drug allergies**	49 (8.2)
**Food allergies**	25 (4.2)
**Type of administered vaccine**	
BNT16b2	
First dose:	446 (74.7)
Second dose:	341 (57.1)
mRNA-1273	
First dose:	151 (25.3)
Second dose:	120 (20.1)
**Humoral immunogenicity (semiquantitative ab titres)**	
Positive	324 (54)
Negative	273 (46)
**IgG titres**	
Positive	164 (99.4)
Negative	2 (1.2)

Covariables of studied population. [a]Diagnosis by RCP, antigen test and antibody positive serology; [b]Number of vaccinees with at least one comorbidity. Results are provided as absolute and relative (%) numbers for each variable

Table S18: Assessment of antibody positiveness (positive-negative titres)

Variable	Antibody positiveness											
	Negative antibody titres (n= 273)						Positive antibody titres (n= 324)					
	**After 1st vaccine dose**	**p-value**	**After 2nd vaccine dose**	**p-value**	**After any dose**	**p-value**	**After 1st vaccine dose**	**p-value**	**After 2nd vaccine dose**	**p-value**	**After any dose**	**p-value**
**Proportions of AR**												
Development of any AR (n, %)	189 (69.2)	0.221	**187 (68.5)**	**0.008**	238 (87.2)	0.445	239 (73.8)	0.221	**188 (58.0)**	**0.008**	289 (89.2)	0.445
Development of any mild AR (n, %)	68 (24.9)	0.296	60 (22.0)	0.100	108 (39.6)	0.115	69 (21.3)	0.296	54 (16.7)	0.100	108 (33.3)	0.115
Development of any moderate AR (n, %)	99 (36.3)	0.099	126 (46.2)	0.052	165 (60.4)	0.690	139 (42.9)	0.099	124 (38.3)	0.052	201 (62.0)	0.690
Development of any severe AR (n, %)	**53 (19.4)**	**0.017**	72 (26.4)	0.411	99 (36.3)	0.204	**90 (27.8)**	**0.017**	76 (23.5)	0.411	134 (41.4)	0.204
Maximum intensity												
Grade 0 (n, %)	**93 (34.1)**	**0.050**	98 (35.9)	0.265	45 (16.5)	0.587	**101 (31.2)**	**0.050**	142 (43.8)	0.265	47 (14.5)	0.587
Grade 1 (n, %)	**48 (17.6)**		26 (9.5)		35 (12.8)		**39 (12.0)**		25 (7.7)		35 (10.8)	
Grade 2 (n, %)	**79 (28.9)**		77 (28.2)		94 (34.4)		**94 (29.0)**		81 (25.0)		108 (33.3)	
Grade 3 (n, %)	**53 (19.4)**		72 (26.4)		99 (36.3)		**90 (27.8)**		76 (23.5)		134 (41.4)	
Likert score Mean (SD)	3.37 (2.99)	0.083	**3.63 (3.31)**	**0.001**	4.93 (2.99)	0.772	3.83 (3.21)	0.083	**3.01 (3.23)**	**0.001**	5.03 (2.95)	0.772

Antibody titres according to proportions (rate) of AR and intensity of AR. AR: adverse reaction; Ab: antibody; in bold type: statistically significant. Results are provided as absolute and relative (%) numbers for each variable

Table S19: Assessment of antibody positiveness (positive-negative titres): solicited and unsolicited AR

**Adverse reactions (PT MedDRA term)**	**Humoral immunogenicity**				**[a]p-value**	
	**Negative antibody titres (n= 273)**		**Positive antibody titres (n= 324)**			
	**After any dose**		**After any dose**			
	**After 1st dose**	**After 2nd dose**	**After 1st dose**	**After 2nd dose**		
**Solicited adverse reactions**						
SOC: *General disorders and administration site conditions*						
Injection site pain *PT: Injection site pain*	**134 (49.1)**		**119 (36.7)**		**0.002**	
	**103 (37.7)**	50 (18.3)	**92 (28.4)**	50 (15.4)	**0.015**	0.347
Fatigue/astenia *PT: Fatigue*	31 (11.4)		23 (7.1)		0.071	
	21 (7.7)	11 (4.0)	20 (6.2)	5 (1.5)	0.465	0.061
Fever *PT: Pyrexia*	91 (33.3)		121 (37.3)		0.308	
	**16 (5.9)**	82 (30.0)	**73 (22.5)**	79 (24.4)	**<0.001**	0.121
Malaise *PT: Malaise*	**37 (13.6)**		**76 (23.5)**		**0.002**	
	**5 (1.8)**	34 (12.5)	**36 (11.1)**	49 (15.1)	**<0.001**	0.348
Chills *PT: Chills*	27 (9.9)		45 (13.9)		0.135	
	**2 (0.7)**	25 (9.2)	**25 (7.7)**	24 (7.4)	**<0.001**	0.438

Adverse reactions (PT MedDRA term)	Humoral immunogenicity				[a]p-value	
	Negative antibody titres (n= 273)		Positive antibody titres (n= 324)			
	After any dose		After any dose			
	After 1st dose	After 2nd dose	After 1st dose	After 2nd dose		
Injection site redness<br>*PT:*<br>*Application site redness*	15 (5.5)		11 (3.4)		0.211	
	10 (3.7)	6 (2.2)	8 (2.5)	3 (0.9)	0.395	0.204
SOC: *Musculoskeletal and connective tissue disorders*						
Arm pain<br>*PT:*<br>*Pain in extremity*	81 (29.7)		90 (27.8)		0.610	
	49 (17.9)	**47 (17.2)**	73 (22.5)	**31 (9.6)**	0.167	**0.006**
Muscle pain<br>*PT:*<br>*Myalgia*	66 (24.2)		81 (25.0)		0.816	
	**9 (3.3)**	58 (21.2)	**36 (11.1)**	55 (17.0)	**0.003**	0.185
Joint pain<br>*PT:*<br>*Arthralgia*	25 (9.2)		27 (8.3)		0.722	
	**0 (0)**	25 (9.2)	**10 (3.1)**	20 (6.2)	**0.034**	0.169
Shoulder pain<br>*PT:*<br>*Musculoskeletal pain*	7 (2.6)		10 (3.1)		0.702	
	5 (1.8)	2 (0.7)	4 (1.2)	5 (1.5)	0.551	0.359
SOC: *Injury, poisoning and procedural complications*						

Adverse reactions (PT MedDRA term)	Humoral immunogenicity				[a]p-value	
	Negative antibody titres (n= 273)		Positive antibody titres (n= 324)			
	After any dose		After any dose			
	After 1st dose	After 2nd dose	After 1st dose	After 2nd dose		
Injection site swelling<br>*PT:*<br>*Application site swelling*	31 (11.4)		23 (7.1)		0.071	
	21 (7.7)	11 (4.0)	20 (6.2)	5 (1.5)	0.465	0.061
SOC: Vaccination site pruritus						
Injection site pruritus<br>*PT:*<br>*Vaccination site pruritus*	8 (2.9)		4 (1.2)		0.141	
	5 (1.8)	3 (1.1)	2 (0.6)	2 (0.6)	0,170	0.520
SOC: Nervous system disorders						
Headache<br>*PT:*<br>*Headache*	79 (28.9)		92 (28.4)		0.884	
	**24 (8.8)**	61 (22.3)	**48 (14.8)**	60 (18.5)	**0.024**	0.247
Facial paralysis<br>*PT:*<br>*Facial paralysis*	0 (0)		0 (0)		NA	
	0 (0)	0 (0)	0 (0)	0 (0)	NA	NA
Insomnia<br>*PT:*<br>*Insomnia*	6 (2.2)		5 (1.5)		0.554	
	1 (0.4)	5 (1.8)	1 (0.3)	4 (1.2)	0.903	0.551
SOC: Gastrointestinal disorders						
Nausea	16 (5.9)		16 (4.9)		0.6181	

**Adverse reactions (PT MedDRA term)**	**Humoral immunogenicity**				**[a]p-value**	
	**Negative antibody titres (n= 273)**		**Positive antibody titres (n= 324)**			
	**After any dose**		**After any dose**			
	**After 1st dose**	**After 2nd dose**	**After 1st dose**	**After 2nd dose**		
PT: Nausea	8 (2.9)	8 (2.9)	11 (3.4)	5 (1.5)	0.747	0.247
Diarrhea	8 (2.9)		9 (2.8)		0.911	
PT: Diarrhoea	3 (1.1)	5 (1.8)	4 (1.2)	5 (1.5)	0.878	0.785
Vomiting	7 (2.6)		3 (0.9)		0.120	
PT: Vomiting	1 (0.4)	6 (2.2)	1 (0.3)	2 (0.6)	0.903	0.094
**Unsolicited adverse reactions**						
SOC: *Musculoskeletal and connective tissue disorders*						
Other musculoskeletal disorders	7 (2.6)		15 (4.6)		0.182	
PT: Musculoskeletal disorder	2 (0.7)	5 (1.8)	9 (2.8)	7 (2.2)	0.064	0.775
SOC: *Skin and subcutaneous tissue disorders*						
Petechia, ecchymosis	0 (0)		1 (0.3)		0.358	
PT: *Ecchymosis*	0 (0)	0 (0)	1 (0.3)	0 (0)	0.358	NA
SOC: *Nervous system disorders*						
Cognitive alteration	0 (0)		0 (0)		NA	
PT: Cognitive disorder	0 (0)	0 (0)	0 (0)	0 (0)	NA	NA
Alterations of smell and taste	1 (0.4)		1 (0.3)		0.903	

Adverse reactions (PT MedDRA term)	Humoral immunogenicity				[a]p-value	
	Negative antibody titres (n= 273)		Positive antibody titres (n= 324)			
	After any dose		After any dose			
	After 1st dose	After 2nd dose	After 1st dose	After 2nd dose		
PT: *Parosmia*	1 (0.4)	0 (0)	0 (0)	1 (0.3)	0.276	0.358
Paresthesia and hyperesthesia	3 (1.1)		4 (1.2)		0.878	
PT: *Dysaesthesia*	1 (0.4)	2 (0.7)	3 (0.9)	1 (0.3)	0.404	0.466
Presyncope, syncope and vasovagal syncope	1 (0.4)		0 (0)		0.276	
PT: *Syncope*	0 (0)	1 (0.4)	0 (0)	0 (0)	NA	0.276
Sleepiness, hypersomnia	0 (0)		0 (0)		NA	
PT: *Hypersomnia*	0 (0)	0 (0)	0 (0)	0 (0)	NA	NA
Instability sensation, vertigo, sickness	6 (2.2)		3 (0.9)		0.204	
PT: *Dizziness*	2 (0.7)	5 (1.8)	2 (0.6)	2 (0.6)	0.863	0.170
Tremor	0 (0)		0 (0)		NA	
PT: *Tremor*	0 (0)	0 (0)	0 (0)	0 (0)	NA	NA
SOC: *Gastrointestinal disorders*						
Gastrointestinal disorders	5 (1.8)		2 (0.6)		0.170	
PT: *Gastrointestinal disorder*	1 (0.4)	**4 (1.5)**	1 (0.3)	**0 (0)**	0.903	**0.029**
SOC: *Respiratory, thoracic and mediastinal disorders*						

**Adverse reactions (PT MedDRA term)**	**Humoral immunogenicity**				**[a]p-value**	
	**Negative antibody titres (n= 273)**		**Positive antibody titres (n= 324)**			
	**After any dose**		**After any dose**			
	**After 1st dose**	**After 2nd dose**	**After 1st dose**	**After 2nd dose**		
Asthma *PT:* *Asthma*	1 (0.4)		0 (0)		0.276	
	1 (0.4)	0 (0)	0 (0)	0 (0)	0.276	NA
Rhinitis, nasal discharge *PT:* *Rhinitis*	2 (0.7)		2 (0.6)		0.863	
	2 (0.7)	0 (0)	1 (0.3)	1 (0.3)	0.466	0.358
SOC: *Investigations*						
Hypertension, hypotension *PT:* *Blood pressure abnormal*	2 (0.7)		0 (0)		0.123	
	0 (0)	2 (0.7)	0 (0)	0 (0)	NA	0.123
SOC: *General disorders and administration site conditions*						
Influenza-like symptoms PT: *Influenza like illness*	5 (1.8)		4 (1.2)		0.551	
	2 (0.7)	4 (1.5)	2 (0.6)	2 (0.6)	0.863	0.301
Sensation of heat, sensation of cold PT: *Temperature regulation disorder*	0 (0)		1 (0.3)		0.358	
	0 (0)	0 (0)	1 (0.3)	0 (0)	0.358	NA

Adverse reactions (PT MedDRA term)	Humoral immunogenicity				[a]p-value	
	Negative antibody titres (n= 273)		Positive antibody titres (n= 324)			
	After any dose		After any dose			
	After 1st dose	After 2nd dose	After 1st dose	After 2nd dose		
Chest pain<br>PT:<br>*Chest pain*	0 (0)		0 (0)		NA	
	0 (0)	0 (0)	0 (0)	0 (0)	NA	NA
Hiporexia, anorexia<br>PT:<br>*Decreased appetite*	0 (0)		0 (0)		NA	
	0 (0)	0 (0)	0 (0)	0 (0)	NA	NA
Inflammation in extremities other than the vaccinated arm<br>PT:<br>*Inflammation*	2 (0.7)		0 (0)		0.123	
	0 (0)	2 (0.7)	0 (0)	0 (0)	NA	0.123
SOC: *Ear and labyrinth disorders*						
PT:<br>*Ear pain*	0 (0)		0 (0)		NA	
	0 (0)	0 (0)	0 (0)	0 (0)	NA	NA
SOC: *Skin and subcutaneous tissue disorders*						
General pruritus<br>PT:<br>*Pruritus*	2 (0.7)		0 (0)		0.123	
	1 (0.4)	0 (0)	1 (0.4)	0 (0)	0.276	0.276
Sweat	2 (0.7)		1 (0.3)		0.466	

Adverse reactions (PT MedDRA term)	Humoral immunogenicity				[a]p-value	
	Negative antibody titres (n= 273)		Positive antibody titres (n= 324)			
	After any dose		After any dose			
	After 1st dose	After 2nd dose	After 1st dose	After 2nd dose		
PT: *Cold sweat*	0 (0)	2 (0.7)	1 (0.3)	0 (0)	0.358	0.123
SOC: *Immune system disorders*						
Immediate hypersensitivity, delayed hypersensitivity, exanthema, urticaria, rash	6 (2.2)		4 (1.2)		0.361	
PT: *Hypersensitivity*	0 (0)	**6 (2.2)**	3 (0.9)	**1 (0.3)**	0.111	**0.033**
SOC: *Cardiac disorders*						
Tachycardia	0 (0)		2 (0.6)		0.194	
PT: *Tachycardia*	0 (0)	0 (0)	1 (0.3)	1 (0.3)	0.358	0.358
SOC: *Blood and lymphatic system disorders*						
Lymphadenopathy	16 (5.9)		9 (2.8)		0.061	
PT: *Lymphadenopathy*	6 (2.2)	11 (4.0)	3 (0.9)	6 (1.9)	0.204	0.111
SOC: *Infections and infestations*						
Herpetic infection	1 (0.4)		1 (0.3)		0.903	
PT: *Herpes virus infection*	1 (0.4)	0 (0)	1 (0.3)	0 (0)	0.903	NA

Distribution of proportions of non-severe solicited and unsolicited AR are displayed by elicited humoral immunogenicity and dose; [a]chi-square test; AR: adverse reaction; SOC: System Organ Class; PT: Preferred term; NA: not applicable, p-value not calculable because of absence of valid values in both comparison groups; in bold type: statistically significant. Results are provided as absolute and relative (%) numbers for each variable

Table S20: Assessment of antibody positiveness (positive-negative titres): severe solicited AR

Adverse reactions (PT MedDRA term)	Humoral immunogenicity				[a]p-value	
	Negative antibody titres (n= 273)		Positive antibody titres (n= 324)			
	After any dose		After any dose			
	After 1st dose	After 2nd dose	After 1st dose	After 2nd dose		
**Solicited adverse reactions**						
SOC: *General disorders and administration site conditions*						
Injection site pain<br>*PT: Injection site pain*	26 (9.5)		30 (9.3)		0.912	
	17 (6.2)	8 (2.9)	20 (6.2)	11 (3.4)	0.978	0.747
Fatigue/astenia<br>*PT:*<br>*Fatigue*	34 (12.5)		40 (12.3)		0.968	
	9 (3.3)	27 (9.9)	14 (4.3)	26 (8.0)	0.517	0.425
Fever<br>*PT:*<br>*Pyrexia*	7 (2.6)		17 (5.2)		0.096	
	**0 (0)**	7 (2.6)	**11 (3.4)**	7 (2.2)	**0.002**	0.746
Malaise<br>*PT:*<br>*Malaise*	**17 (6.2)**		**42 (13.0)**		**0.006**	
	**3 (1.1)**	14 (5.1)	**18 (5.6)**	26 (8.0)	**0.003**	0.159
Injection site redness<br>*PT:*<br>*Application site redness*	5 (1.7)		2 (0.6)		0.170	
	4 (1.4)	1 (0.3)	2 (0.6)	0 (0)	0.301	0.276

**Adverse reactions (PT MedDRA term)**	**Humoral immunogenicity**				**[a]p-value**	
	**Negative antibody titres** (n= 273)		**Positive antibody titres** (n= 324)			
	**After any dose**		**After any dose**			
	**After 1st dose**	**After 2nd dose**	**After 1st dose**	**After 2nd dose**		
SOC: *Musculoskeletal and connective tissue disorders*						
Arm pain *PT: Pain in extremity*	28 (10.3)		35 (10.8)		0.829	
	19 (7.0)	11 (4.0)	28 (8.6)	8 (2.5)	0.447	0.279
Muscle pain *PT: Myalgia*	23 (8.0)		36 (11.7)		0.273	
	**3 (1.0)**	20 (6.9)	**18 (5.8)**	21 (6.8)	**0.003**	0.684
Joint pain *PT: Arthralgia*	14 (5.1)		17 (5.2)		0.948	
	**0 (0)**	14 (5.1)	**6 (1.9)**	13 (4.0)	**0.024**	0.513
Shoulder pain *PT: Musculoskeletal pain*	1 (0.4)		2 (0.6)		0.666	
	1 (0.4)	0 (0)	1 (0.3)	0 (0)	0.903	NA
SOC: *Injury, poisoning and procedural complications*						
Injection site swelling *PT: Application site swelling*	9 (3.3)		9 (2.8)		0.712	
	8 (2.9)	1 (0.4)	8 (2.5)	1 (0.3)	0.728	0.903
SOC: Vaccination site pruritus						
Injection site pruritus *PT: Vaccination site pruritus*	0 (0)		1 (0.3)		0.358	
	0 (0)	0 (0)	1 (0.3)	0 (0)	0.358	NA

Adverse reactions (PT MedDRA term)	Humoral immunogenicity				[a]p-value	
	Negative antibody titres (n= 273)		Positive antibody titres (n= 324)			
	After any dose		After any dose			
	After 1st dose	After 2nd dose	After 1st dose	After 2nd dose		
SOC: Nervous system disorders						
Headache	24 (8.8)		40 (12.3)		0.162	
PT: Headache	**6 (2.2)**	19 (7.0)	**20 (6.2)**	21 (6.5)	**0.018**	0.816

Distribution of proportions of severe solicited and unsolicited AR are displayed by elicited humoral immunogenicity and dose; [a]chi-square test; AR: adverse reaction; SOC: System Organ Class; PT: Preferred term; NA: not applicable, p-value not calculable because of absence of valid values in both comparison groups; in bold type: statistically significant. Results are provided as absolute and relative (%) numbers for each variable

Table S21: Assessment of antibody titres (antibody titres above-below median):

Variable	Antibody titres below median* (n= 288)						Antibody titres above median* (n= 309)					
	**After 1st vaccine dose**	**p-value**	**After 2nd vaccine dose**	**p-value**	**After any dose**	**p-value**	**After 1st vaccine dose**	**p-value**	**After 2nd vaccine dose**	**p-value**	**After any dose**	**p-value**
**Proportions of AR**												
Development of any AR (n, %)	200 (69.4)	0.239	**193 (67.0)**	**0.040**	249 (86.5)	0.183	228 (73.8)	0.239	**182 (58.9)**	**0.040**	278 (90.0)	0.183
Development of any mild AR (n, %)	70 (24.3)	0.446	62 (21.5)	0.144	111 (38.5)	0.247	67 (21.7)	0.446	52 (16.8)	0.144	105 (34.0)	0.247
Development of any moderate AR (n, %)	**103 (35.8)**	**0.048**	129 (44.8)	0.163	172 (59.7)	0.443	**135 (43.7)**	**0.048**	121 (39.2)	0.163	194 (62.8)	0.443
Development of any severe AR (n, %)	60 (20.8)	0.085	75 (26.0)	0.494	107 (37.2)	0.364	83 (26.9)	0.085	73 (23.6)	0.494	126 (40.8)	0.364
Maximum intensity												
Grade 1 (n, %)	50 (26.2)	0.072	27 (14.9)	0.935	36 (15.1)	0.719	37 (17.5)	0.072	24 (13.6)	0.935	34 (12.8)	0.719
Grade 2 (n, %)	81 (42.4)		79 (43.6)		96 (40.2)		92 (43.4)		79 (44.9)		106 (39.8)	
Grade 3 (n, %)	60 (31.4)		75 (41.4)		107 (44.8)		83 (39.2)		73 (41.5)		126 (47.4)	
Likert score<br>N<br>Mean (SD)<br>Median (P25, P75)<br>min,Max	 189<br>5.28 (2.18)<br>5.00 (3.00, 7.00)<br>{1.00, 10.00}	0.083	 175<br>5.87 (2.13)<br>6.00 (4.00, 8.00)<br>{2.00,10.00}	0.570	 236<br>6.06 (2.15)<br>6.00 (4.00, 8.00)<br>{1.00,10.00}	0.592	 206<br>5.65 (2.09)<br>6.00 (4.00, 7.00)<br>{0.00,10.00}	0.083	 163<br>5.77 (2.02)<br>6.00 (4.00, 7.00)<br>{2.00,10.00}	0.570	 259<br>5.97 (2.07)<br>6.00 (4.00, 8.00)<br>{0.00,10.00}	0.592

Antibody titres (in titres above and below median) according to proportions (rate) of AR and intensity of AR. AR: adverse reaction; Ab: antibody; in bold type: statistically significant results. Results are provided as absolute and relative (%) numbers for each variable, Median value of antibody titres (P25, P75)= 2.53 (0.47 , 10.00) on a semiquantitative scale ranging from 0 to >10

Table S22: Assessment of antibody titres (antibody titres above-below median): solicited and unsolicited AR

**Adverse reactions (PT MedDRA term)**	**Humoral immunogenicity**				**[a]p-value**	
	**Antibody titres below median* (n= 288)**		**Antibody titres above median* (n= 309)**			
	**After any dose**		**After any dose**			
	**After 1st dose**	**After 2nd dose**	**After 1st dose**	**After 2nd dose**		
**Solicited adverse reactions**						
SOC: *General disorders and administration site conditions*						
Injection site pain *PT: Injection site pain*	**141 (49.0)**		**112 (36.2)**		**0.002**	
	**110 (38.2)**	52 (18.1)	**85 (27.5)**	48 (15.5)	**0.005**	0.410
Fatigue/astenia *PT: Fatigue*	74 (25.7)		86 (27.8)		0.556	
	**24 (8.3)**	58 (20.1)	**43 (13.9)**	53 (17.2)	**0.031**	0.350
Fever *PT: Pyrexia*	95 (33.0)		117 (37.9)		0.213	
	**19 (6.6)**	83 (28.8)	**70 (22.7)**	78 (25.2)	**<0.001**	0.325
Malaise *PT: Malaise*	**41 (14.2)**		**72 (23.3)**		**0.004**	
	**7 (2.4)**	37 (12.8)	**34 (11.0)**	46 (14.9)	**<0.001**	0.472
Chills *PT: Chills*	29 (10.1)		43 (13.9)		0.149	
	**4 (1.4)**	25 (8.7)	**23 (7.4)**	24 (7.8)	**0.004**	0.685

Adverse reactions (PT MedDRA term)	Humoral immunogenicity				[a]p-value	
	**Antibody titres below median* (n= 288)**		**Antibody titres above median* (n= 309)**			
	**After any dose**		**After any dose**			
	**After 1st dose**	**After 2nd dose**	**After 1st dose**	**After 2nd dose**		
Injection site redness	15 (5.2)		11 (3.6)		0.324	
PT: Application site redness	10 (3.5)	6 (2.1)	8 (2.6)	3 (1.0)	0.528	0.265
SOC: *Musculoskeletal and connective tissue disorders*						
Arm pain	85 (29.5)		86 (27.8)		0.650	
PT: Pain in extremity	52 (18.1)	49 (17.0)	70 (22.7)	29 (9.4)	0.164	0.006
Muscle pain	68 (23.6)		79 (25.6)		0.580	
PT: Myalgia	**9 (3.1)**	60 (20.8)	**36 (11.7)**	53 (17.2)	**<0.001**	0.251
Joint pain	28 (9.7)		24 (7.8)		0.397	
PT: Arthralgia	**1 (0.3)**	27 (9.4)	**9 (2.9)**	18 (5.8)	**0.015**	0.101
Shoulder pain	9 (3.1)		8 (2.6)		0.694	
PT: Musculoskeletal pain	6 (2.1)	3 (1.0)	3 (1.0)	4 (1.3)	0.265	0.774
SOC: *Injury, poisoning and procedural complications*						
Injection site swelling	31 (10.8)		23 (7.4)		0.158	
PT: Application site swelling	21 (7.3)	11 (3.8)	20 (6.5)	5 (1.6)	0.693	0.096
SOC: Vaccination site pruritus						
Injection site pruritus	8 (2.8)		4 (1.3)		0.200	
PT:	5 (1.7)	3 (1.0)	2 (0.6)	2 (0.6)	0.217	0.597

Adverse reactions (PT MedDRA term)	Humoral immunogenicity				ᵃp-value	
	Antibody titres below median* (n= 288)		Antibody titres above median* (n= 309)			
	After any dose		After any dose			
	After 1st dose	After 2nd dose	After 1st dose	After 2nd dose		
Vaccination site pruritus						
SOC: Nervous system disorders						
Headache	83 (28.8)		88 (28.5)		0.927	
PT: Headache	**26 (9.0)**	64 (22.2)	**46 (14.9)**	57 (18.4)	**0.028**	0.252
Facial paralysis	0 (0)		0 (0)		NA	
PT: Facial paralysis	0 (0)	0 (0)	0 (0)	0 (0)	NA	NA
Insomnia	6 (2.1)		5 (1.6)		0.673	
PT: Insomnia	1 (0.3)	5 (1.7)	1 (0.3)	4 (1.3)	0.960	0.658
SOC: Gastrointestinal disorders						
Nausea	17 (5.9)		15 (4.9)		0.570	
PT: Nausea	9 (3.1)	8 (2.8)	10 (3.2)	5 (1.6)	0.938	0.332
Diarrhea	9 (3.1)		8 (2.6)		0.694	
PT: Diarrhoea	3 (1.0)	6 (2.1)	4 (1.3)	4 (1.3)	0.774	0.453
Vomiting	7 (2.4)		3 (1.0)		0.165	

Adverse reactions (PT MedDRA term)	Humoral immunogenicity				[a]p-value	
	Antibody titres below median* (n= 288)		Antibody titres above median* (n= 309)			
	After any dose		After any dose			
	After 1st dose	After 2nd dose	After 1st dose	After 2nd dose		
PT: Vomiting	1 (0.3)	6 (2.1)	1 (0.3)	2 (0.6)	0.960	0.127
**Unsolicited adverse reactions**						
SOC: *Musculoskeletal and connective tissue disorders*						
Other musculoskeletal disorders	8 (2.8)		14 (4.5)		0.256	
PT: Musculoskeletal disorder	3 (1.0)	5 (1.7)	8 (2.6)	7 (2.3)	0.160	0.645
SOC: *Skin and subcutaneous tissue disorders*						
Petechia, ecchymosis	0 (0)		1 (0.3)		0.334	
PT: *Ecchymosis*	0 (0)	0 (0)	1 (0.3)	0 (0)	0.334	NA
SOC: *Nervous system disorders*						
Cognitive alteration	0 (0)		0 (0)		NA	
PT: Cognitive disorder	0 (0)	0 (0)	0 (0)	0 (0)	NA	NA
Alterations of smell and taste	1 (0.3)		1 (0.3)		0.960	
PT: Parosmia	1 (0.3)	0 (0)	0 (0)	1 (0.3)	0.300	0.334
Paresthesia and hyperesthesia	3 (1.0)		4 (1.3)		0.774	

**Adverse reactions (PT MedDRA term)**	**Humoral immunogenicity**				**[a]p-value**	
	**Antibody titres below median* (n= 288)**		**Antibody titres above median* (n= 309)**			
	**After any dose**		**After any dose**			
	**After 1st dose**	**After 2nd dose**	**After 1st dose**	**After 2nd dose**		
PT: Dysaesthesia	1 (0.3)	2 (0.7)	3 (1.0)	1 (0.3)	0.351	0.522
Presyncope, syncope and vasovagal syncope	1 (0.3)		0 (0)		0.300	
PT: Syncope	0 (0)	1 (0.3)	0 (0)	0 (0)	NA	0.300
Sleepiness, hypersomnia	0 (0)		0 (0)		NA	
PT: Hypersomnia	0 (0)	0 (0)	0 (0)	0 (0)	NA	NA
Instability sensation, vertigo, sickness	6 (2.1)		3 (1.0)		0.265	
PT: Dizziness	2 (0.7)	5 (1.7)	2 (0.6)	2 (0.6)	0.944	0.217
Tremor	0 (0)		0 (0)		NA	
PT: Tremor	0 (0)	0 (0)	0 (0)	0 (0)	NA	NA
SOC: *Gastrointestinal disorders*						
Gastrointestinal disorders	**6 (2.1)**		**1 (0.3)**		**0.046**	
PT: Gastrointestinal disorder	1 (0.3)	**4 (1.4)**	1 (0.3)	**0 (0)**	0.960	**0.038**
SOC: *Respiratory, thoracic and mediastinal disorders*						

Adverse reactions (PT MedDRA term)	Humoral immunogenicity				[a]p-value	
	Antibody titres below median* (n= 288)		Antibody titres above median* (n= 309)			
	After any dose		After any dose			
	After 1st dose	After 2nd dose	After 1st dose	After 2nd dose		
Asthma *PT: Asthma*	1 (0.3)		0 (0)		0.300	
	1 (0.3)	0 (0)	0 (0)	0 (0)	0.300	NA
Rhinitis, nasal discharge *PT: Rhinitis*	2 (0.7)		2 (0.6)		0.944	
	2 (0.7)	0 (0)	1 (0.3)	1 (0.3)	0.522	0.334
SOC: *Investigations*						
Hypertension, hypotension *PT: Blood pressure abnormal*	2 (0.7)		0 (0)		0.142	
	0 (0)	2 (0.7)	0 (0)	0 (0)	NA	0.142
SOC: *General disorders and administration site conditions*						
Influenza-like symptoms PT: *Influenza like illness*	5 (1.7)		4 (1.3)		0.658	
	2 (0.7)	4 (1.4)	2 (0.6)	2 (0.6)	0.944	0.364
Sensation of heat, sensation of cold PT: *Temperature regulation disorder*	0 (0)		1 (0.3)		0.334	
	0 (0)	0 (0)	1 (0.3)	0 (0)	0.334	NA

**Adverse reactions (PT MedDRA term)**	**Humoral immunogenicity**				**[a]p-value**	
	**Antibody titres below median* (n= 288)**		**Antibody titres above median* (n= 309)**			
	**After any dose**		**After any dose**			
	**After 1st dose**	**After 2nd dose**	**After 1st dose**	**After 2nd dose**		
Chest pain PT:*Chest pain*	0 (0)		0 (0)		NA	
	0 (0)	0 (0)	0 (0)	0 (0)	NA	NA
Hiporexia, anorexia PT: *Decreased appetite*	0 (0)		0 (0)		NA	
	0 (0)	0 (0)	0 (0)	0 (0)	NA	NA
Inflammation in extremities other than the vaccinated arm PT: *Inflammation*	2 (0.7)		0 (0)		0.142	
	0 (0)	2 (0.7)	0 (0)	0 (0)	NA	0.142
SOC: *Ear and labyrinth disorders*						
PT: *Ear pain*	0 (0)		0 (0)		NA	
	0 (0)	0 (0)	0 (0)	0 (0)	NA	NA
SOC: *Skin and subcutaneous tissue disorders*						
General pruritus PT: *Pruritus*	**2 (0.7)**		**0 (0)**		**0.142**	
	1 (0.4)	1 (0.4)	0 (0)	0 (0)	0.300	0.300

**Adverse reactions (PT MedDRA term)**	**Humoral immunogenicity**				**[a]p-value**	
	**Antibody titres below median* (n= 288)**		**Antibody titres above median* (n= 309)**			
	**After any dose**		**After any dose**			
	**After 1st dose**	**After 2nd dose**	**After 1st dose**	**After 2nd dose**		
Sweat PT: *Cold sweat*	2 (0.7)		1 (0.3)		0.522	
	0 (0)	2 (0.7)	1 (0.3)	0 (0)	0.334	0.142
SOC: *Immune system disorders*						
Immediate hypersensitivity, delayed hypersensitivity, exanthema, urticaria, rash PT: *Hypersensitivity*	6 (2.1)		4 (1.3)		0.453	
	0 (0)	**6 (2.1)**	3 (1.0)	**1 (0.3)**	0.094	**0.046**
SOC: *Cardiac disorders*						
Tachycardia PT: *Tachycardia*	0 (0)		2 (0.6)		0.171	
	0 (0)	0 (0)	1 (0.3)	1 (0.3)	0.334	0.334
SOC: *Blood and lymphatic system disorders*						
Lymphadenopathy PT: *Lymphadenopathy*	16 (5.6)		9 (2.9)		0.107	
	6 (2.1)	11 (3.8)	3 (1.0)	6 (1.9)	0.265	0.168
SOC: *Infections and infestations*						
Herpetic infection PT:	1 (0.3)		1 (0.3)		0.960	
	1 (0.3)	0 (0)	1 (0.3)	0 (0)	0.960	NA

Adverse reactions (PT MedDRA term)	Humoral immunogenicity				[a]p-value	
	Antibody titres below median* (n= 288)		Antibody titres above median* (n= 309)			
	After any dose		After any dose			
	After 1st dose	After 2nd dose	After 1st dose	After 2nd dose		
Herpes virus infection						

Distribution of proportions of non-severe solicited and unsolicited AR are displayed by elicited humoral immunogenicity and dose; [a]chi-square test; AR: adverse reaction; SOC: System Organ Class; PT: Preferred term; NA: not applicable, p-value not calculable because of absence of valid values in both comparison groups; in bold type: statistically significant results. Results are provided as absolute and relative (%) numbers for each variable, Median value of antibody titres (P25, P75)= 2.53 (0.47 , 10.00) on a semiquantitative scale ranging from 0 to >10

Table S23: Assessment of antibody titres (antibody titres above-below median): severe solicited AR

**Adverse reactions (PT MedDRA term)**	**Humoral immunogenicity**				**[a]p-value**	
	**Humoral immunogenicity**		**Humoral immunogenicity**			
	**Antibody titres below median* (n= 288)**		**Antibody titres above median* (n= 309)**			
	**After any dose**	**After any dose**	**After any dose**	**After any dose**		
**Solicited adverse reactions**						
SOC: *General disorders and administration site conditions*						
Injection site pain	31 (10.8)		25 (8.1)		0.263	
PT: Injection site pain	22 (7.6)	8 (2.8)	15 (4.9)	11 (3.6)	0.159	0.587
Fatigue/astenia	35 (12.2)		39 (12.6)		0.862	
PT: Fatigue	10 (3.5)	27 (9.4)	13 (4.2)	26 (8.4)	0.641	0.680
Fever	8 (2.8)		16 (5.2)		0.136	
PT: Pyrexia	**1 (0.3)**	7 (2.4)	**10 (3.2)**	7 (2.3)	**0.009**	0.894
Malaise	**19 (6.6)**		**40 (12.9)**		**0.009**	
PT: Malaise	**5 (1.7)**	15 (5.2)	**16 (5.2)**	25 (8.1)	**0.023**	0.159
Injection site redness	5 (1.7)		2 (0.6)		0.217	
PT: Application site redness	4 (1.4)	1 (0.3)	2 (0.6)	0 (0)	0.364	0.300
SOC: *Musculoskeletal and connective tissue disorders*						
Arm pain	30 (10.4)		33 (10.7)		0.917	

Adverse reactions (PT MedDRA term)	Humoral immunogenicity				[a]p-value	
	Humoral immunogenicity		Humoral immunogenicity			
	Antibody titres below median* (n= 288)		Antibody titres above median* (n= 309)			
	After any dose	After any dose	After any dose	After any dose		
PT: Pain in extremity	20 (6.9)	12 (4.2)	27 (8.7)	7 (2.3)	0.416	0.186
Muscle pain	23 (8.0)		36 (11.7)		0.134	
PT: Myalgia	**3 (1.0)**	20 (6.9)	**18 (5.8)**	21 (6.8)	**0.002**	0.943
Joint pain	16 (5.6)		15 (4.9)		0.700	
PT: Arthralgia	1 (0.3)	15 (5.2)	5 (1.6)	12 (3.9)	0.120	0.436
Shoulder pain	2 (0.7)		1 (0.3)		0.522	
PT: Musculoskeletal pain	2 (0.7)	0 (0)	0 (0)	0 (0)	0.142	NA
SOC: *Injury, poisoning and procedural complications*						
Injection site swelling	9 (3.1)		9 (2.9)		0.880	
PT: Application site swelling	8 (2.8)	1 (0.3)	8 (2.6)	1 (0.3)	0.887	0.960
SOC: Vaccination site pruritus						
Injection site pruritus	0 (0)		1 (0.3)		0.334	
PT: Vaccination site pruritus	0 (0)	0 (0)	1 (0.3)	0 (0)	0.334	NA
SOC: Nervous system disorders						
Headache	26 (9.0)		38 (12.3)		0.200	

Adverse reactions (PT MedDRA term)	Humoral immunogenicity				[a]p-value	
	Humoral immunogenicity		Humoral immunogenicity			
	Antibody titres below median* (n= 288)		Antibody titres above median* (n= 309)			
	After any dose	After any dose	After any dose	After any dose		
PT: Headache	**6 (2.1)**	21 (7.3)	**20 (6.5)**	19 (6.1)	**0.009**	0.577

Distribution of proportions of severe solicited and unsolicited AR are displayed by elicited humoral immunogenicity and dose; [a]chi-square test; AR: adverse reaction; SOC: System Organ Class; PT: Preferred term; NA: not applicable, p-value not calculable because of absence of valid values in both comparison groups; in bold type: statistically significant results. Results are provided as absolute and relative (%) numbers for each variable, Median value of antibody titres (P25, P75)= 2.53 (0.47 , 10.00) on a semiquantitative scale ranging from 0 to >10

Figure S3: Summary of results of immunogenicity and reactogenicity of sensitivity analysis 2

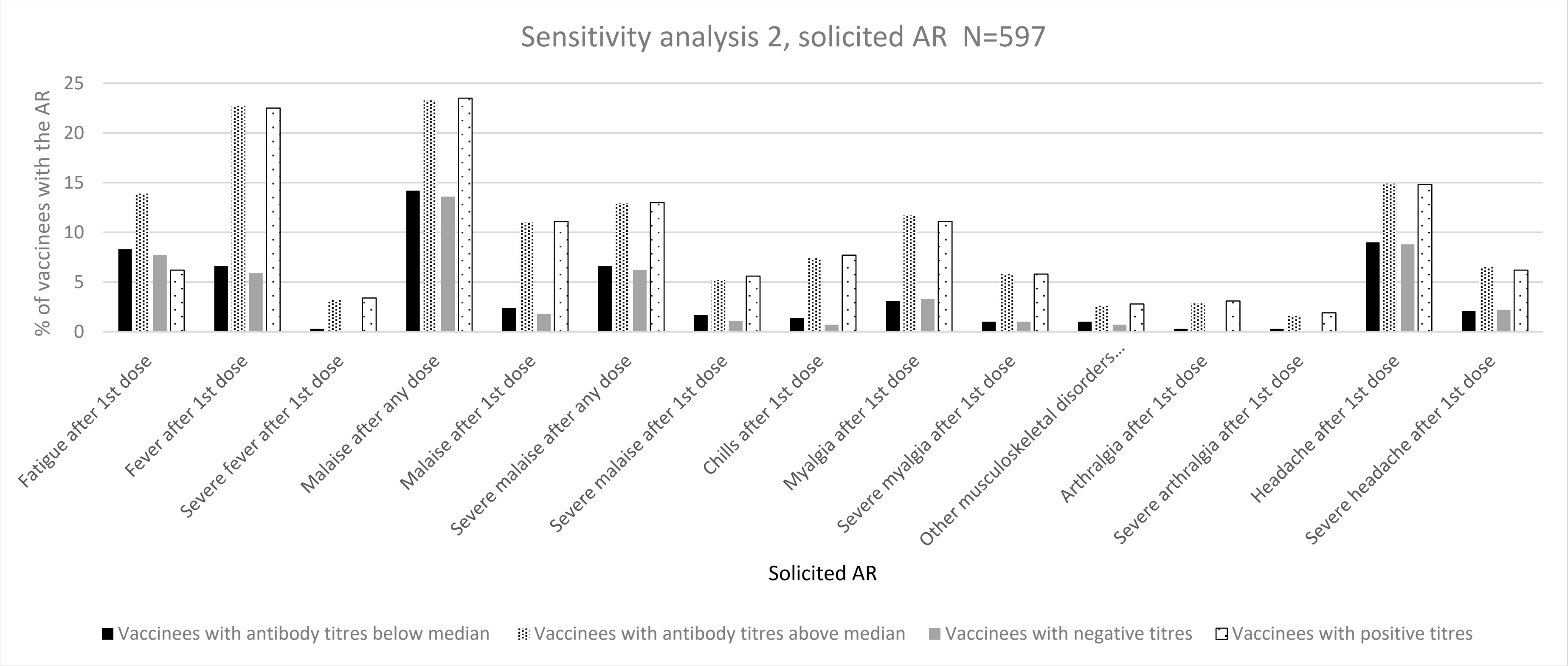

Bar diagram of results of main analysis. Immunogenicity is expressed as antibody positiveness (positive-negative titres) and cellular immunogenicity (positive-negative) data (%). Reactogenicity results are represented as % of vaccinees developing each solicited AR

Table S24: Reactogenicity and immunogenicity by vaccine and population types

Subpopulation 1		Vaccine type (mRNA-1273 vs BNT162b2)		Population type (HCP vs SOTR)		HCP		SOTR	
N=3563		mRNA-1273 All vaccinees N=865	BNT162b2 All vaccinees N=2698	HCP N=3460	SOTR N=103	HCP vaccinated with mRNA-1273 N=762	HCP vaccinated with BNT162b2 N=2698	SOTR vaccinated with mRNA-1273 N=103	SOTR vaccinated with BNT162b2
**Immunogenicity (n, %)**									
Qualitative immunogenicity	Positive titres	810 (94)	2677 (99)	3428 (99)	59 (57)	746 (98.5)	2665 (99)	59 (57)	NA
	Negative titres	55 (6)	21 (1)	32 (1)	44 (43)	11 (1.5)	21 (1)	44 (43)	NA
**Reactogenicity (n, %)**									
Any AR after any dose		832 (96.2)	2203 (81.7)	2945 (85.1)	90 (87.4)	742 (97.4)	2203 (81.7)	90 (87.4)	NA
Any severe AR after any dose		500 (57.8)	922 (34.2)	1393 (40.3)	29 (28.2)	471 (61.8)	922 (34.2)	29 (28.2)	NA
Injection site pain		431 (49.8)	952 (35.3)	1334 (38.6)	49 (47.6)	382 (50.1)	952 (35.3)	49 (47.6)	NA
Fatigue/astenia		292 (33.8)	713 (26.4)	984 (28.4)	21 (20.4)	271 (35.6)	713 (26.4)	21 (20.4)	NA
Fever		439 (50.8)	673 (24.9)	1090 (31.5)	22 (21.4)	417 (54.7)	673 (24.9)	22 (21.4)	NA
Malaise		212 (24.5)	417 (15.5)	621 (17.9)	8 (7.8)	204 (26.8)	417 (15.5)	8 (7.8)	NA
Injection site redness		77 (8.9)	80 (3.0)	155 (4.5)	2 (1.9)	75 (9.8)	80 (3.0)	2 (1.9)	NA
Arm pain		341 (39.4)	731 (27.1)	1040 (30.1)	32 (31.1)	309 (40.6)	731 (27.1)	32 (31.1)	NA
Myalgia		191 (22.1)	523 (19.4)	707 (20.4)	7 (6.8)	184 (24.1)	523 (19.4)	7 (6.8)	NA
Arthralgia		114 (13.2)	218 (8.1)	329 (9.5)	3 (2.9)	111 (14.6)	218 (8.1)	3 (2.9)	NA
Shoulder pain		46 (5.3)	68 (2.5)	104 (3.0)	10 (9.7)	36 (4.7)	68 (2.5)	10 (9.7)	NA
Injection site swelling		120 (13.9)	131 (4.9)	245 (7.1)	6 (5.8)	114 (15.0)	131 (4.9)	6 (5.8)	NA
Injection site pruritus		49 (5.7)	24 (0.9)	70 (2.0)	3 (2.9)	46 (6.0)	24 (0.9)	3 (2.9)	NA
Headache		297 (34.3)	681 (25.2)	963 (27.8)	15 (14.6)	282 (37.0)	681 (25.2)	15 (14.6)	NA

HCP: health-care professional; SOTR: solid-organ transplant recipient; NA: not applicable because all SOTR were vaccinated with mRNA-1273

Figure S4: Comparison of vaccine types

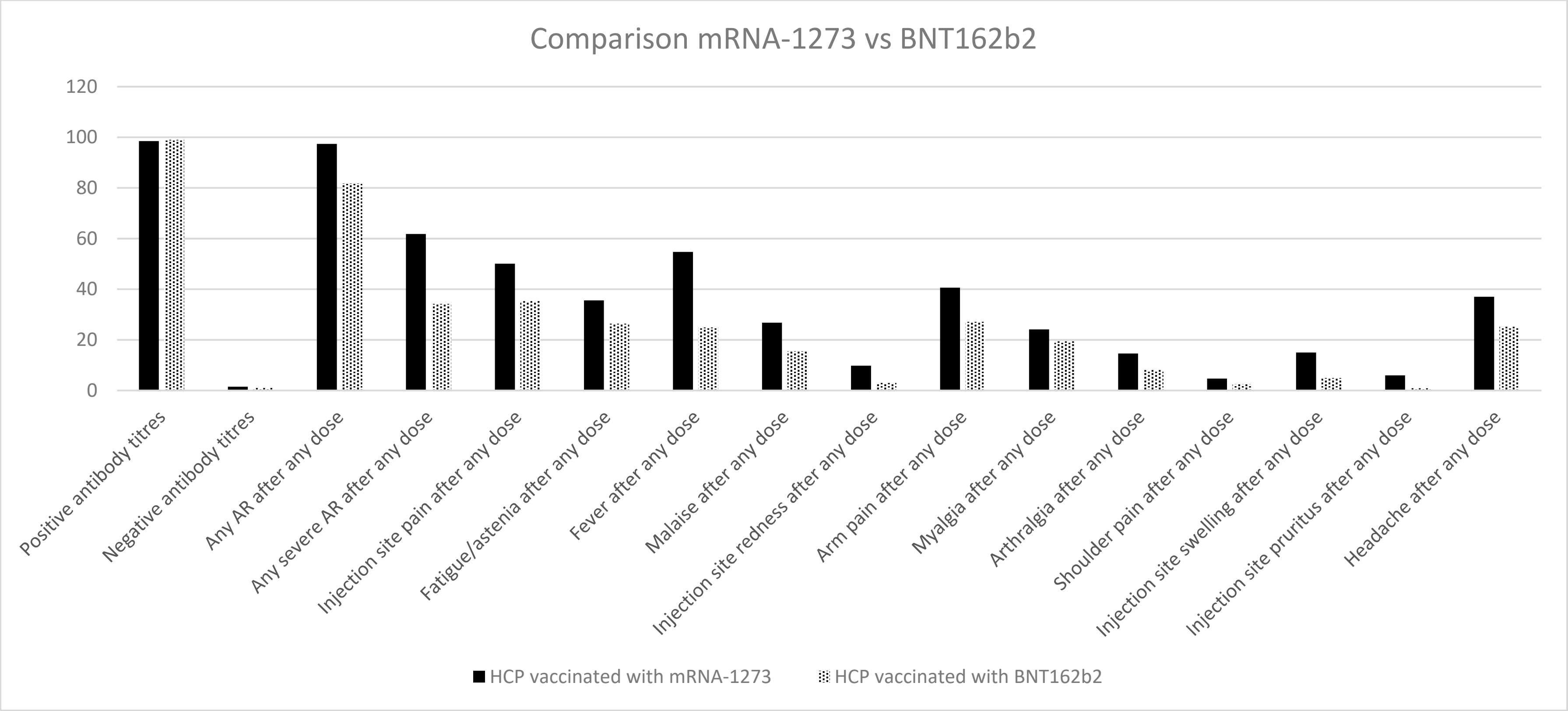

HCP: health-care professional; SOTR: solid-organ transplant recipient; AR: adverse reaction; positive/negative antibody titres: % of vaccinees with positive or negative titres; any AR, any severe AR and solicited AR: % of vaccines with at least one event

Figure S5: Comparison of population types

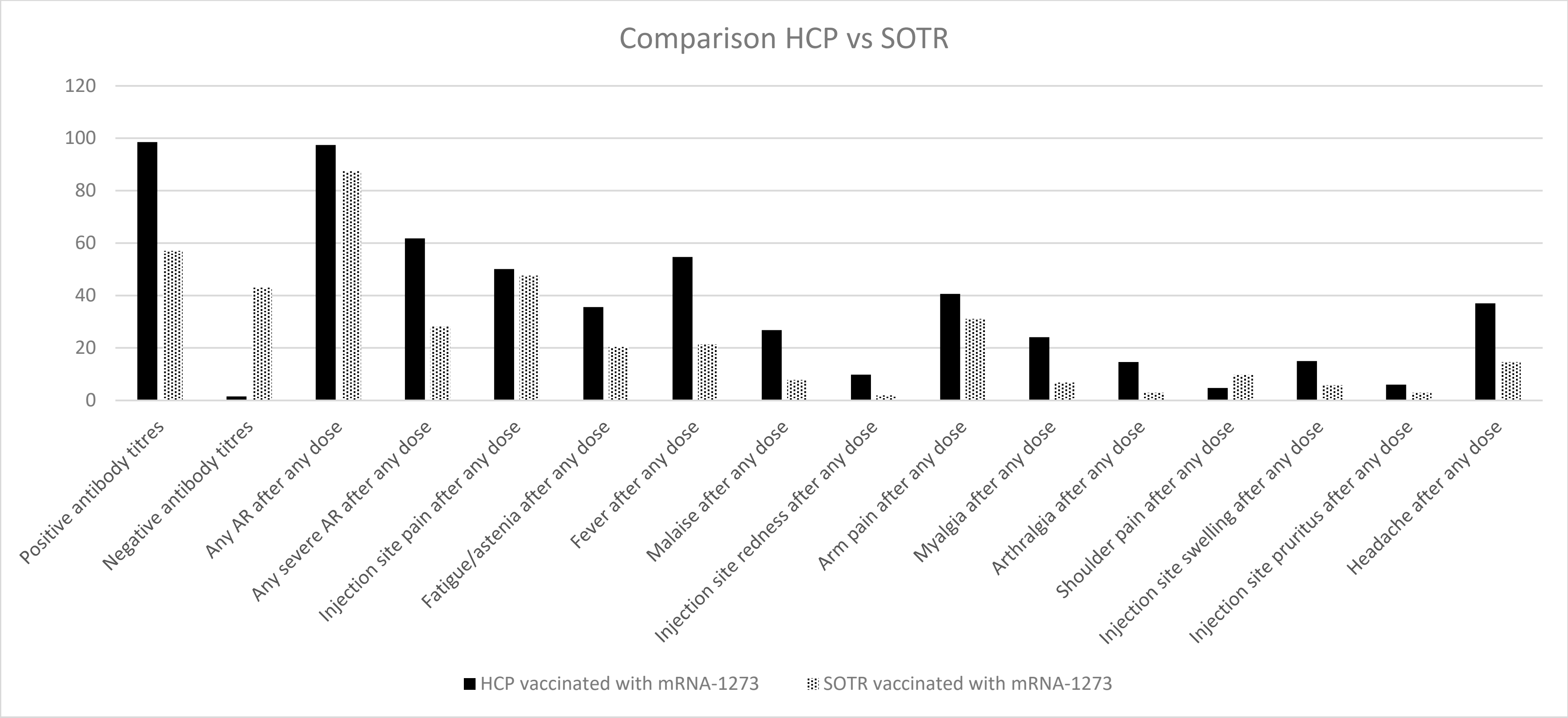

HCP: health-care professional; SOTR: solid-organ transplant recipient; AR: adverse reaction; positive/negative antibody titres: % of vaccinees with positive or negative titres; any AR, any severe AR and solicited AR: % of vaccines with at least one event

Table S25 STROBE Checklist

STROBE Statement—Checklist of items that should be included in reports of ***cross-sectional studies***

	Item No	Recommendation	Page No*
**Title and abstract**	1	(*a*) Indicate the study's design with a commonly used term in the title or the abstract	1
		(*b*) Provide in the abstract an informative and balanced summary of what was done and what was found	2
**Introduction**			
Background/rationale	2	Explain the scientific background and rationale for the investigation being reported	4-5
Objectives	3	State specific objectives, including any prespecified hypotheses	7-8
**Methods**			
Study design	4	Present key elements of study design early in the paper	5
Setting	5	Describe the setting, locations, and relevant dates, including periods of recruitment, exposure, follow-up, and data collection	5-6
Participants	6	(*a*) Give the eligibility criteria, and the sources and methods of selection of participants	5-6
Variables	7	Clearly define all outcomes, exposures, predictors, potential confounders, and effect modifiers. Give diagnostic criteria, if applicable	7-8
Data sources/ measurement	8*	For each variable of interest, give sources of data and details of methods of assessment (measurement). Describe comparability of assessment methods if there is more than one group	7-9
Bias	9	Describe any efforts to address potential sources of bias	7
Study size	10	Explain how the study size was arrived at	6
Quantitative variables	11	Explain how quantitative variables were handled in the analyses. If applicable, describe which groupings were chosen and why	7-9
Statistical methods	12	(*a*) Describe all statistical methods, including those used to control for confounding	7-9
		(*b*) Describe any methods used to examine subgroups and interactions	

		(*c*) Explain how missing data were addressed		
		(*d*) If applicable, describe analytical methods taking account of sampling strategy		
		(*e*) Describe any sensitivity analyses		
**Results**				
Participants	13*	(a) Report numbers of individuals at each stage of study—eg numbers potentially eligible, examined for eligibility, confirmed eligible, included in the study, completing follow-up, and analysed	10	
		(b) Give reasons for non-participation at each stage		
		(c) Consider use of a flow diagram		
Descriptive data	14*	(a) Give characteristics of study participants (eg demographic, clinical, social) and information on exposures and potential confounders	10	
		(b) Indicate number of participants with missing data for each variable of interest		
Outcome data	15*	Report numbers of outcome events or summary measures	10-12	
Main results	16	(*a*) Give unadjusted estimates and, if applicable, confounder-adjusted estimates and their precision (eg, 95% confidence interval). Make clear which confounders were adjusted for and why they were included	10-12	
		(*b*) Report category boundaries when continuous variables were categorized		
		(*c*) If relevant, consider translating estimates of relative risk into absolute risk for a meaningful time period		
Other analyses	17	Report other analyses done—eg analyses of subgroups and interactions, and sensitivity analyses	10-12	

**Discussion**			
Key results	18	Summarise key results with reference to study objectives	13-15
Limitations	19	Discuss limitations of the study, taking into account sources of potential bias or imprecision. Discuss both direction and magnitude of any potential bias	14-15
Interpretation	20	Give a cautious overall interpretation of results considering objectives, limitations, multiplicity of analyses, results from similar studies, and other relevant evidence	12-16
Generalisability	21	Discuss the generalisability (external validity) of the study results	15-16
**Other information**			
Funding	22	Give the source of funding and the role of the funders for the present study and, if applicable, for the original study on which the present article is based	3

*Pages where the different items of the STROBE checklist are described

Reference: https://www.strobe-statement.org/
